# Structure–activity analysis of imino‐pyrimidinone‐fused pyrrolidines aids the development of dual plasmepsin V and plasmepsin X inhibitors

**DOI:** 10.1111/febs.70038

**Published:** 2025-03-04

**Authors:** Anthony N. Hodder, Brad E. Sleebs, Greg Adams, Sina Rezazadeh, Anna Ngo, Kate Jarman, Stephen Scally, Peter Czabotar, Hongwu Wang, John A. McCauley, David B. Olsen, Alan F. Cowman

**Affiliations:** ^1^ The Walter and Eliza Hall Institute of Medical Research Parkville Australia; ^2^ Department of Medical Biology The University of Melbourne Parkville Australia; ^3^ Merck & Co., Inc. West Point PA USA

**Keywords:** dual inhibition, imino‐pyrimidinone‐fused pyrrolidine, plasmepsin IX, plasmepsin V, plasmepsin X

## Abstract

A library of known aspartic protease inhibitors was screened to identify compounds that inhibit plasmepsin V from *Plasmodium vivax*. This screen revealed compounds with an imino‐pyrimidinone‐fused pyrrolidine (IPF) scaffold that exhibited sub‐micromolar inhibitory activity against plasmepsin V. Further screening of IPF analogs against the related aspartic protease plasmepsin X showed inhibitory activity, while a third aspartic protease, plasmepsin IX, was not significantly inhibited. Modifications to the P1 biaryl region of the IPF scaffold differentially modulated inhibition of both plasmepsin V and X. Notably, analogs with potent plasmepsin X inhibitory activity successfully blocked the growth of *Plasmodium falciparum in vitro*. X‐ray structures of IPF analogs in complex with plasmepsin V provided insights into their binding mode and revealed avenues to further improve IPF potency and selectivity between plasmepsin V and X. This understanding of how these compounds interact with the active sites of plasmepsin V and X will serve as a foundation for the future design of dual inhibitors targeting these proteases.

## Graphical abstract

Compounds were identified that inhibit the aspartic protease plasmepsin V from *Plasmodium vivax*. Further development of these compounds identified compounds, such as S‐WM48, that could inhibit both plasmepsin V and plasmepsin X but plasmepsin IX was not significantly inhibited. X‐ray structures of S‐WM48 in complex with plasmepsin V provided insights into their binding mode and revealed avenues to further improve IPF potency and selectivity between plasmepsin V and X.

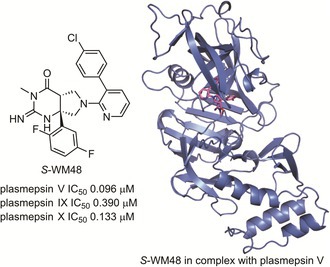

**Abbreviations**
ACTs: artemisinin combination therapies
GIA: growth inhibition assay
IPF: imino‐pyrimidinone‐fused pyrrolidine
KAHRP: knob associated histidine‐rich protein
PEXEL: *Plasmodium* export element
PMIX: plasmepsin IX
PMV: plasmepsin V
PMX: plasmepsin X
VTS: vacuolar targeting sequence

## Introduction

Malaria is a major disease of humans caused by parasites from *Plasmodium* spp. *P. falciparum* is the deadliest form causing more than 90% of deaths due to malaria worldwide. *Plasmodium vivax* is endemic to South‐East Asia and the Americas and responsible for 30% of infections worldwide. *P. vivax* is of concern as it has a dormant liver stage that can result in recrudescence of symptomatic malaria months after the initial infection or drug treatment. RTS,S/AS01 is an approved vaccine against *P. falciparum* infection, but it is only 35% effective [1]. Promisingly, the R21 Matrix‐M vaccine in clinical trials has shown up to 70% effectiveness [2]. Small‐molecule therapies have historically been the mainstay in the prevention and treatment of malaria, and artemisinin combination therapies (ACTs) are the current first‐line antimalarials. Worryingly, an overreliance on a narrow spectrum of antimalarials chemotypes has resulted in widespread resistance against most antimalarials, including ACTs, underscoring the need for the development of a pipeline for new drug candidates [3, 4].

High‐throughput screening of large compound libraries against *P. falciparum* has unearthed a plethora of new chemical classes for the development of antimalarials, and this has resulted in several promising candidates that are currently in clinical trials [5, 6]. Concerningly, drug‐resistant parasites have been isolated from drug‐treated volunteers for some of these novel drugs [5], highlighting the need for antimalarials with a high barrier against the development of drug resistance.

A protein class that has emerged from these approaches are the aspartyl proteases. *P. falciparum* expresses 10 cathepsin D‐like aspartyl proteases, known as plasmepsins. Plasmepsins I–IV are localized to the digestive vacuole of the parasite and involved in degrading hemoglobin to provide sustenance metabolites for parasite development, but none of these proteases are essential for parasite growth [7, 8]. Genetic disruption of the genes encoding plasmepsin I, II, IV, and histo‐aspartyl protease (HAP) in a single‐parasite strain showed only a slight defect in growth rate [7, 8]. Plasmepsins VI–VIII are expressed in the mosquito stages, and their roles remain undefined. Evidence suggests plasmepsins VI and VIII are essential for *Plasmodium berghei* development in the mosquito, while plasmepsin VII appears to be redundant and nonessential [9, 10, 11].

Plasmepsin IX and X (PMIX and PMX) have high structural homology and are both indispensable for asexual parasite development [12, 13, 14]. The subcellular localization of PMX has been identified, and it appears to be in the micronemes and exonemes. This protease proteolytically processes invasion ligands and proteins required for egress. In contrast, PMIX has been localized to the rhoptries, and it proteolytically matures a smaller repertoire of proteins [13, 14, 15]. PMIX and X proteolytic activity has been shown to be important at other stages of the parasite life cycle. In the transmission stage, they are required for oocyte formation, although their precise roles are currently unknown [14]. PMX is also crucial for the proteolytic maturation of invasion ligands in the merozoite of the liver stage schizont, allowing the merozoite to invade the host erythrocyte and establish the asexual blood stage infection [14]. Peptidomimetic‐like compounds such as 49c and drug‐like compounds including WM382 and UCB7362 have been described that inhibit either PMIX or PMX [13, 14, 16, 17]. WM382 has been designed to be a dual inhibitor of both PMIX and X [14, 16]. X‐ray structures and modeling of these compounds in complex with PMIX and PMX have aided in understanding their binding mode and selectivity for inhibition of PMIX and X to support their development as antimalarial candidates [18].

Plasmepsin V (PMV) is essential for the development of the asexual and sexual stage parasites and plays a crucial role in the export of proteins from the parasite cytosol to the host red blood cell [19, 20, 21, 22]. Exported proteins have an important role in solute‐waste exchange from the erythrocyte, evasion of the host immune system, and sequestration of hemoglobin from the red blood cell for sustenance [23, 24, 25]. Many proteins that are exported have a five amino acid N‐terminal motif, with the consensus sequence RxLxQ/E/D, known as *Plasmodium* export element (PEXEL) or the vacuolar targeting sequence (VTS) [26, 27]. The PEXEL motif is processed on the C‐terminal side of leucine by plasmepsin V, which targets the mature protein for export to the red blood cell [19, 20, 23]. The PEXEL motif and PMV are highly conserved across all *Plasmodium* spp., suggesting that this protease is a promising essential antimalarial drug target.

**Fig. 1:**
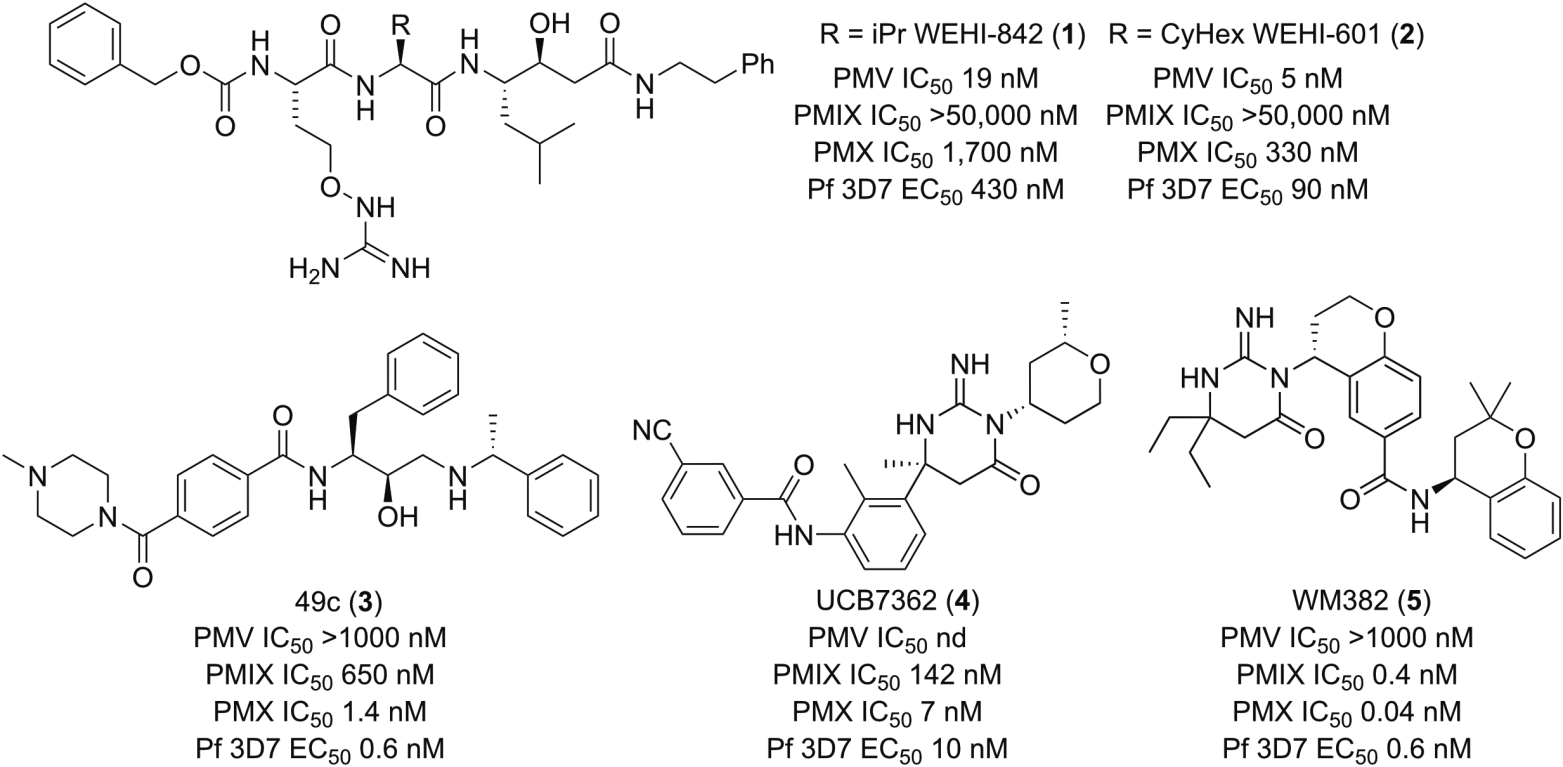
Structures and biological activities of plasmepsin V, IX, and X inhibitors. Shown are the plasmepsin aspartic protease activity (IC_50_) for PMV, PMIX, and PMX. Also shown is the blood stage inhibitory growth activity (EC_50_) of each compound against the 3D7 strain of *Plasmodium falciparum* 3D7 obtained from the literature [17, 18, 21, 33, 34].

Peptidomimetics that mimic the PEXEL substrate including WEHI‐842 and WEHI‐601 (Fig. 1) have been used to inhibit PMV and demonstrate its druggability and importance in asexual and sexual stage parasite development [21, 22, 28, 29, 30]. X‐ray structures of these peptidomimetics in complex with PMV have led to a better understanding of substrate specificity and the inhibitor binding mode, aiding further development of these compounds as antimalarials [29].

WEHI‐842 and WEHI‐601 have modest activity against the asexual parasite despite their potent biochemical inhibition of PMV (Fig. 1), which was thought to be attributed to their low membrane permeability [29, 30]. However, recent evidence suggests that PMV is highly expressed, and therefore, concentrations of an inhibitor more than its enzymatic IC_50_ value may be required to enable efficient killing of the malaria parasite [31]. A further limitation of using peptidomimetics to target PMV is their low metabolic stability, which hinders their development as antimalarial therapeutics. Drug‐like inhibitors of the plasmepsin proteases have previously been identified using a high‐throughput screen against *P. falciparum* of an aspartyl protease inhibitor library [17]. Hit compounds identified were potent inhibitors of PMX, and from these starting points, WM382 was identified, and it potently targets both PMIX and PMX [14, 16] (Fig. 1). Additionally, the small‐molecule UCB7362 (Fig. 1) has been identified, and it more specifically targets PMX [17]. To date, no drug‐like compounds have been discovered that potently target PMV.

To uncover nonpeptidomimetic or drug‐like inhibitors of PMV, a library of compounds was screened that target human aspartyl proteases [32, 33, 34], against *P. vivax* PMV. A hit compound WM36 was identified that inhibited PMV protease activity; however, it did not inhibit *P. falciparum* blood stage development. The structure of WM36 consists of an imino‐pyrimidinone‐fused pyrrolidine scaffold (Table 1), herein referred to as IPF. IPF analogs with a variety of substitutions and modifications to the biphenyl moiety were evaluated for inhibition of PMV, PMIX, and PMX to determine their structure–activity relationship and selectivity profile between plasmepsins. X‐ray structures of WM36 and IPF analogs bound to PMV were obtained to understand their binding mode and specific interactions with amino acids in binding pockets. The small structural modifications to the IPF scaffold modulated potency and selectivity between plasmepsins providing insight into plasmepsin inhibitor design.

**Table 1:** Activity of analogs with substitution on the P_1_′ aryl group.

R corresponds to the structure:					
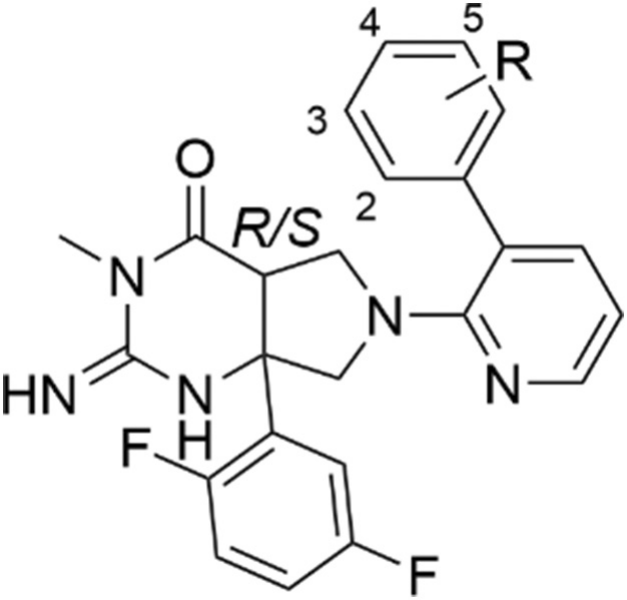					
IC_50_ data represent averages and SD for three independent fluorogenic substrate cleavage experiments with recombinant *Plasmodium vivax* PMV, *Plasmodium falciparum* PMIX or *P. falciparum* PMX					
EC_50_ data represent averages and SD for three independent experiments measuring LDH activity of *P. falciparum* 3D7 asexual parasites following exposure to compounds for 72 h					
Compound	R	PMV	PMIX	PMX	Pf 3D7
		IC_50_ (SD), μm	IC_50_ (SD), μm	IC_50_ (SD), μm	EC_50_ (SD), μm
**6**	H	0.440 (0.105)	3.00 (0.27)	1.07 (0.12)	> 10
**7**	2‐F	1.02 (0.23)	4.53 (0.70)	1.33 (0.06)	> 10
*R/S*‐WM36 (**8**)	2‐Cl	0.807 (0.078)	4.87 (0.76)	1.10 (< 0.01)	> 10
**9**	2‐Me	0.610 (0.114)	5.50 (0.72)	2.07 (0.72)	> 10
**10**	2‐CF_3_	3.45 (0.50)	3.97 (0.83)	1.28 (0.68)	> 10
**11**	2‐OMe	0.303 (0.032)	> 10	7.50 (3.04)	> 10
**12**	3‐Cl	> 3.7	0.560 (0.044)	0.390 (0.066)	> 10
**13**	3‐OMe	1.50 (0.26)	1.17 (0.15)	0.473 (0.045)	> 10
*R/S*‐WM48 (**14**)	4‐Cl	0.357 (0.061)	1.08 (0.14)	0.343 (0.023)	> 10
**15**	4‐OMe	3.60 (0.42)	2.00 (0.17)	0.473 (0.085)	> 10
**16**	2‐F, 4‐Cl	0.780 (0.017)	0.417 (0.038)	1.33 (0.15)	> 10
**17**	2‐Cl, 4‐Cl	0.517 (0.025)	0.360 (0.017)	1.07 (0.15)	> 10
**18**	2‐CF_3_, 4‐Cl	> 3.7	1.08 (0.14)	0.483 (0.038)	> 10
**19**	3‐Cl, 4‐Cl	> 3.7	0.434 (0.086)	0.187 (0.072)	> 10
**20**	3‐F, 4‐Cl	1.01 (0.16)	1.15 (0.18)	0.440 (0.035)	> 10
**21**	2‐Cl, 4‐F	0.967 (0.221)	3.23 (0.50)	0.990 (0.010)	> 10
**22**	2‐Cl, 4‐Me	0.690 (0.078)	1.63 (0.21)	0.420 (0.010)	> 10
**23**	2‐Cl, 4‐OMe	3.17 (1.07)	1.63 (0.41)	0.420 (0.027)	> 10

## Results

### Identification of inhibitors for the aspartic protease *Pv*PMV

To uncover nonpeptidomimetic drug‐like inhibitors of the aspartic protease PvPMV, we screened a library of 1298 compounds that were known to inhibit human aspartyl proteases [32, 33, 34]. These compounds were selected by chemoinformatic structural diversity from a larger library of compounds that targeted human aspartyl proteases and has been used previously to identify hit compounds that inhibited *P. falciparum* growth [14]. These hit compounds belonged to a single chemical class and were used to develop a lead compound WM382 that is a dual selective inhibitor of PMIX and PMX from *P. falciparum* and *P. vivax* [14, 16]. The screen utilized a FRET‐based assay with PvPMV and a fluorogenic EDANS‐DACBYL labeled peptide with the *P. falciparum* knob‐associated histidine‐rich protein (KAHRP) PEXEL motif amino acid sequence [14]. This screen identified the hit compound WM36 and a dose–response assay against PMV confirmed the inhibitory activity with an IC_50_ of 0.807 μm (Table 1).

Using a biochemical assay that utilized fluorogenic peptides mimicking the amino acid sequences of the proteins RON3 and Rh2N substrates of PMIX and PMX, respectively [14], WM36 was shown to inhibit both PMIX and PMX with IC_50_ values of 4.87 and 1.10 μm respectively. WM36 did not inhibit *P. falciparum* asexual parasite replication consistent with the parasite activity of other compounds with modest inhibition of plasmepsins [28, 30, 35].

The structure of WM36 consists of an imino‐pyrimidinone‐fused pyrrolidine scaffold (Table 1), herein referred to as IPF. The imino‐pyrimidinone (IP) head group was known to engage with two aspartic acids in the catalytic dyad to elicit inhibitory activity [36]. The IPF scaffold was decorated with a substituted biaryl moiety on the pyrrolidine nitrogen and a substituted phenyl group at the carbon bridgehead. These substituted groups on the IPF scaffold are known to occupy the S_1_ and S_2_′/S_1_′ pockets, respectively, which provides the selectivity between aspartyl proteases [32].

### Structure–activity relationship of IPF analogs against PMV, PMIX and PMX

IPF analogs with a variety of substitutions and modifications to the biphenyl moiety were evaluated for inhibitory activity against PMV, PMIX, and PMX to determine their structure–activity relationship and selectivity profile between these plasmepsins. Notably, the hit compound, WM36 (**8**), was a mixture of *R/S*‐isomers at the carbon bridgehead of the IPF scaffold (Table 1). Therefore, the analogs that were sourced for exploration of the SAR were also a mixture of *R/S*‐isomers.

SAR exploration of the IPF scaffold primarily focused on modifications to the biphenyl ring that was engaged with the S_1_ and S_2_′ pockets of the plasmepsin proteases because it was reasoned that changes to this motif would most likely affect inhibitory selectivity. The IP head group and the 2,5‐difluoro substituted ring that occupies the S_1_ pocket remained unchanged for this study. Examination of the SAR began with point modifications with small functional groups on the terminal aryl ring of the biphenyl motif to determine whether these changes are favorable or deleterious for the inhibition of PMV, PMIX, or PMX. It was shown that deleting the 2‐chloro substituent (**6**) gave a modest improvement in PMV inhibition (IC_50_ 0.440 μm) compared with WM36 (**8**) (IC_50_ 0.807 μm) (Table 1). Installation of a 2‐fluoro (**7**) or a 2‐methyl (**9**) had no significant effect on the inhibitory activity (IC_50_ 1.02 and 0.610 μm), while a 2‐trifluoromethyl group (**10**) reduced activity (IC_50_ 3.45 μm) and a 2‐methoxy group (**11**) improved PMV inhibition by 2.5‐fold (IC_50_ 0.303 μm). Iterations in the 2‐position had no significant bearing on PMIX or PMX inhibitory activity. The introduction of a 3‐chloro or 3‐methoxy (**12** and **13**) both decreased PMV activity (IC_50_ > 3.7 and 1.50 μm) but did enhance both PMIX and PMX activity (IC_50_ 0.390–1.17 μm). The incorporation of a 4‐chloro (WM48, **14**) enhanced PMV activity (IC_50_ 0.357 μm) but a larger 4‐methoxy group (**15**) decreased PMV activity (IC_50_ 3.60 μm). While these 4‐position iterations did not change PMIX activity (IC_50_ 1.08 and 2.00 μm), PMX activity was enhanced (IC_50_ 0.343 and 0.473 μm).

We next sought to introduce combinations of two substituents on the terminal aryl ring of the biphenyl moiety to determine the effect on plasmepsin protease activity. The inclusion of a second substituent on the terminal aryl ring may give an additional interaction with amino acids in the S_1_ pocket leading to enhanced inhibition of PMV, PMIX, or PMX. This first combination of analogs had a 4‐chloro while altering the 2‐position with a small functional group. The addition of a 2‐fluoro or a 2‐chloro (**16** and **17**) resulted in an approximate twofold loss in PMV inhibition (IC_50_ 0.780 and 0.517 μm) relative to the activity of WM48 (**14**) (IC_50_ 0.357 μm) (Table 1). The PMX activity of analogs **16** and **17** was unchanged (IC_50_ 1.33 and 1.07 μm), whereas the PMIX potency (IC_50_ 0.417 and 0.360 μm) was like the activity of WM48 (**14**). The introduction of a larger trifluoromethyl group in the 2‐position (**18**) decreased the ability of this compound to inhibit PMV activity (IC_50_ > 3.7 μm), but anti‐PMX inhibition was maintained (IC_50_ 0.483 μm). The inclusion of 3‐chloro or 3‐fluoro in combination with a 4‐chloro (**19** and **20**) was detrimental to PMV inhibition (IC_50_ > 3.7 and 1.01 μm), consistent with the activity of the analogs **12** and **13** with the mono substitution in 3‐position. The analog **19** with a 3,4‐dichloro substitution pattern modestly increased the PMX potency (IC_50_ 0.187 μm). Altering the 4‐position to a fluoro, methyl, or methoxy while maintaining a 2‐chloro substituent (**21**, **22** and **23**) did not improve the PMV inhibition (IC_50_ 0.690–3.17 μm) or alter PMIX (IC_50_ 1.63–3.23 μm) or PMX activity (IC_50_ 0.420–0.990 μm) relative to the activity of WM48 against these PMs. The analogs from Table 1 did not exhibit antiparasitic activity in the *P. falciparum* asexual stage assay (EC_50_ > 10 μm). It was reasoned that the plasmepsin inhibitory activity of these analogs was not adequately potent to affect parasite growth.

Analogs were next explored that replaced the terminal aryl ring on the biphenyl system with a thiophene. The introduction of a thiophene ring would slightly alter the substituent vector bond angle compared with substitution on the aryl ring, which could be favorable for the inhibition of PMV, PMIX, or PMX or selectivity between these proteases. It was found that the unsubstituted thiophene variation (**24**) decreased PMV inhibition (IC_50_ 1.23 μm) relative to WM48 (**14**), but strikingly enhanced PMX inhibition (IC_50_ 0.067 μm), while PMIX activity was unchanged (IC_50_ 1.20 μm) (Table 2). The introduction of a 5‐chloro substituent on the thiophene (**26**) did not alter PMX activity (IC_50_ 0.070 μm), although modestly increased PMV activity (IC_50_ 0.610 μm). The inclusion of a 3‐chloro substituent (WM396, **25**) further improved the PMX activity by eightfold (IC_50_ 0.009 μm), without altering PMV inhibition (IC_50_ 1.11 μm) and modestly improving PMIX inhibition (IC_50_ 0.350 μm). These data suggest that PMV has a larger S_1_ pocket to accommodate the 4‐substituted phenyl ring, while PMX has a shallow S_1_ pocket that was more suited to accommodating a thiophene ring. The thiophene‐derived analogs (**24**–**26**) exhibited measurable antiparasitic activity against asexual stage *P. falciparum* parasites (EC_50_s' 0.200–2.50 μm). The antiparasitic activity was proportional to the PMX inhibitory activity, and WM396 showed the most potent antimalarial activity (EC_50_ 0.2 μm). These data suggested that PMX was the primary driver of antiparasitic activity and not PMV or PMIX.

**Table 2:** Activity of analogs with substitution on the P_1_′ thiophene.

R refers to the following structure:					
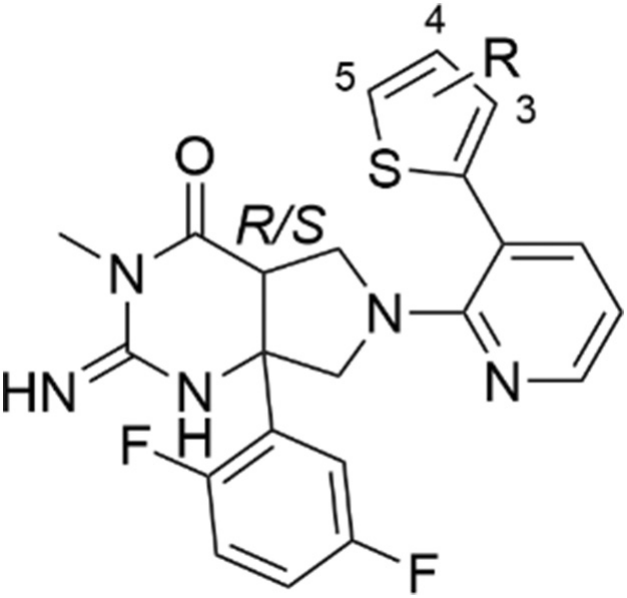					
IC_50_ data represent averages and SD for three independent fluorogenic substrate cleavage experiments with recombinant *Plasmodium vivax* PMV, *Plasmodium falciparum* PMIX or *P. falciparum* PMX					
EC_50_ data represent averages and SD for three independent experiments measuring LDH activity of *P. falciparum* 3D7 asexual parasites following exposure to compounds for 72 h					
Compound	R	PMV	PMIX	PMX	Pf 3D7
		IC_50_ (SD), μm	IC_50_ (SD), μm	IC_50_ (SD), μm	EC_50_ (SD), μm
**24**	H	1.23 (0.15)	1.20 (< 0.01)	0.067 (0.003)	1.77 (0.06)
*R/S*‐WM396 (**25**)	3‐Cl	1.11 (0.18)	0.350 (0.037)	0.009 (0.002)	0.200 (0.018)
**26**	5‐Cl	0.610 (0.010)	–	0.070	2.50 (0.01)

We next examined bioisosteric replacements for the P_2_′ pyridyl ring of the biphenyl moiety. The introduction of different 5‐membered heteroaromatic systems, while maintaining the position of the endocyclic nitrogen, may alter the vector bond angle of substituents or the inclusion of another endocyclic nitrogen which could be beneficial for binding to PMV, PMIX, or PMX. It was revealed that replacing the pyridine ring with a thiazole (**27**), the PMV and PMIX inhibitory activity was unchanged compared with analog **6**, while the PMX inhibition was enhanced by twofold (IC_50_ 0.473 μm) (Table 3). Exchanging the pyridyl ring for a pyrazole (WM447, **28**) led to a further eightfold improvement in PMX activity (IC_50_ 0.064 μm), while the PMV and PMIX activity was maintained. The PMX activity (IC_50_ 3.47 μm) could be reversed by N‐methyl substitution of the pyrazole (**29**), signifying the pyrazole N–H on analog WM447 (**28**) could be forming a hydrogen bond interaction with an amino acid in the S_1_ pocket of PMX that was absent in PMV and PMIX. The unsubstituted pyrazole analog WM447 (**28**) exhibited modest antimalarial activity (EC_50_ 1.53 μm), while the other bioisosteric analogs did not have significant antimalarial activity, consistent with our previous observation (with analogs **24**–**26**) that PMX was the primary driver of antimalarial activity.

**Table 3:** Activity of P_2_′ 2‐pyridyl bioisostere analogs.

R refers to the following structure:					
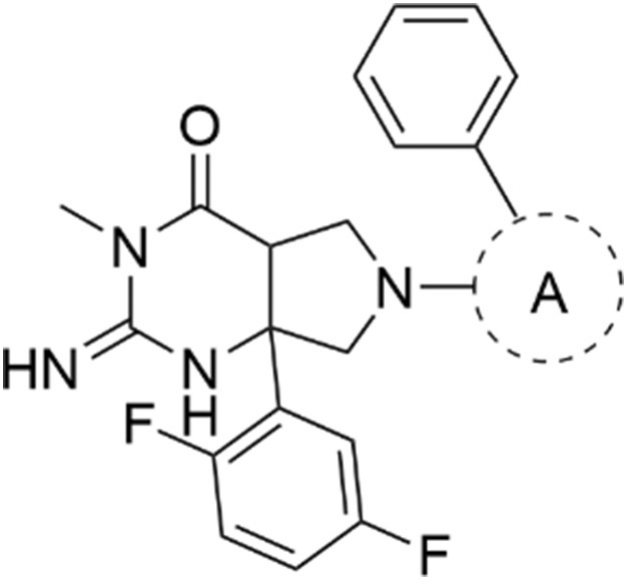					
Compound	A^a^	PMV	PMIX	PMX	Pf 3D7
		IC_50_ (SD), μm ^b^	IC_50_ (SD), μm ^b^	IC_50_ (SD), μm ^b^	EC_50_ (SD), μm ^c^
**6**	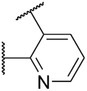	0.440 (0.105)	3.00 (0.26)	1.07 (0.15)	> 10
**27**	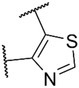	0.440 (0.061)	0.990 (0.116)	0.473 (0.031)	> 10
*R/S*‐WM447 (**28**)	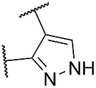	0.683 (0.038)	2.17 (0.35)	0.064 (0.001)	1.53 (0.12)
**29**	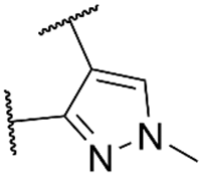	1.05 (0.09)	10.4 (1.4)	3.47 (0.06)	> 10

^a^ A refers to the functional group above surrounded by a dotted circle.

^b^ IC_50_ data represent averages and SD for three independent fluorogenic substrate cleavage experiments with recombinant *Plasmodium vivax* PMV, *Plasmodium falciparum* PMIX or *P. falciparum* PMX.

^c^ EC_50_ data represent averages and SD for three independent experiments measuring LDH activity of *P. falciparum* 3D7 asexual parasites following exposure to compounds for 72 h.

**Fig. 2:**
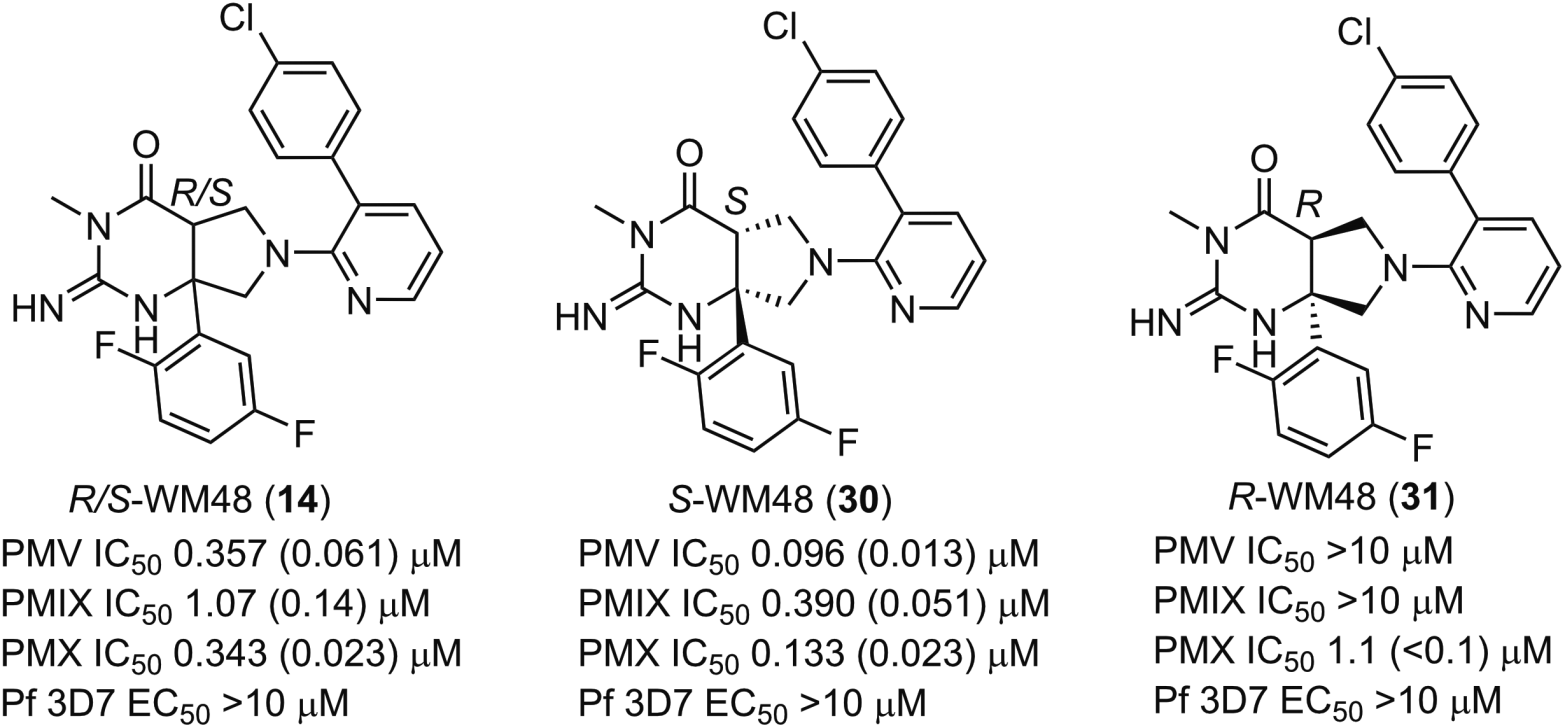
Structures of the racemic mixture (R/S), S and R‐stereoisomers of WM48 and inhibition of the proteases PMV, PMIX, and PMX (IC_50_) and *Plasmodium falciparum* blood stage growth (EC_50_). Data are averages (SDs) of *n* = 3 experiments.

All analogs investigated in the study (Tables 1, 2, 3) were a racemic mixture of *R‐* and *S‐* isomers at the carbon bridgehead of the IPF scaffold. The fused pyrrolidone group for each isomer would be orientated in a different direction, and therefore, one isomer should be preferred for binding to the S_1_′ and S_1_ pocket of the plasmepsins. To determine whether the stereochemistry at the bridgehead was important for activity, the stereoisomers of *R/S*‐WM48 (**14**) were separated by chiral chromatography. The fractionated isomers expectedly displayed differential activity against the plasmepsins (Fig. 2), but the absolute stereochemistry of the active and less active fractions was unknown. The stereochemistry of the active isomer was determined by subjecting *R/S*‐WM48 (**14**) to crystallization trials with PMV. The *S‐*WM48 (**30**) isomer (Fig. 2) crystallized in the presence of PMV (see next section), and it was reasoned that it was the most potent isomer. This was further supported by obtaining an X‐ray structure of the *S‐*isomer of WM36 in complex with PMV (see next section).

It was observed that the *S‐*WM48 (**30**) exhibited inhibition of PMV, PMIX, and PMX (IC_50_ 0.096, 0.390, and 0.133 μm), while *R*‐WM48 (**31**) had significantly reduced activity against PMV, PMIX, and PMX (IC_50_ > 10, > 10, and 1.0 μm) (Fig. 2). *S‐*WM48 (**30**) did not exhibit antimalarial activity at the concentration tested (EC_50_ > 10 μm). The PMX inhibitory activity of *S*‐WM48 (**14**) was not sufficiently potent to register antiparasitic activity at concentrations below 10 μm, whereas compounds **24**–**26** that exhibit inhibition of PMX (IC_50_ < 0.10 μm) registered antiparasitic activity (Table 2). Moreover, the level of PMV inhibition by *S*‐WM48 was not adequate to register antiparasitic activity. In comparison, WEHI‐842 (**1**) and WEHI‐601 (**2**) that exhibit inhibition of PMV (IC_50_ < 0.02 μm) exhibit antiparasitic activity (Fig. 1). Collectively, these data suggest that the antiparasitic activity observed with analogs **24**–**26** and **28** is derived from the inhibition of PMX.

### Structural analysis of IPF analogs bound to PMV

**Fig. 3:**
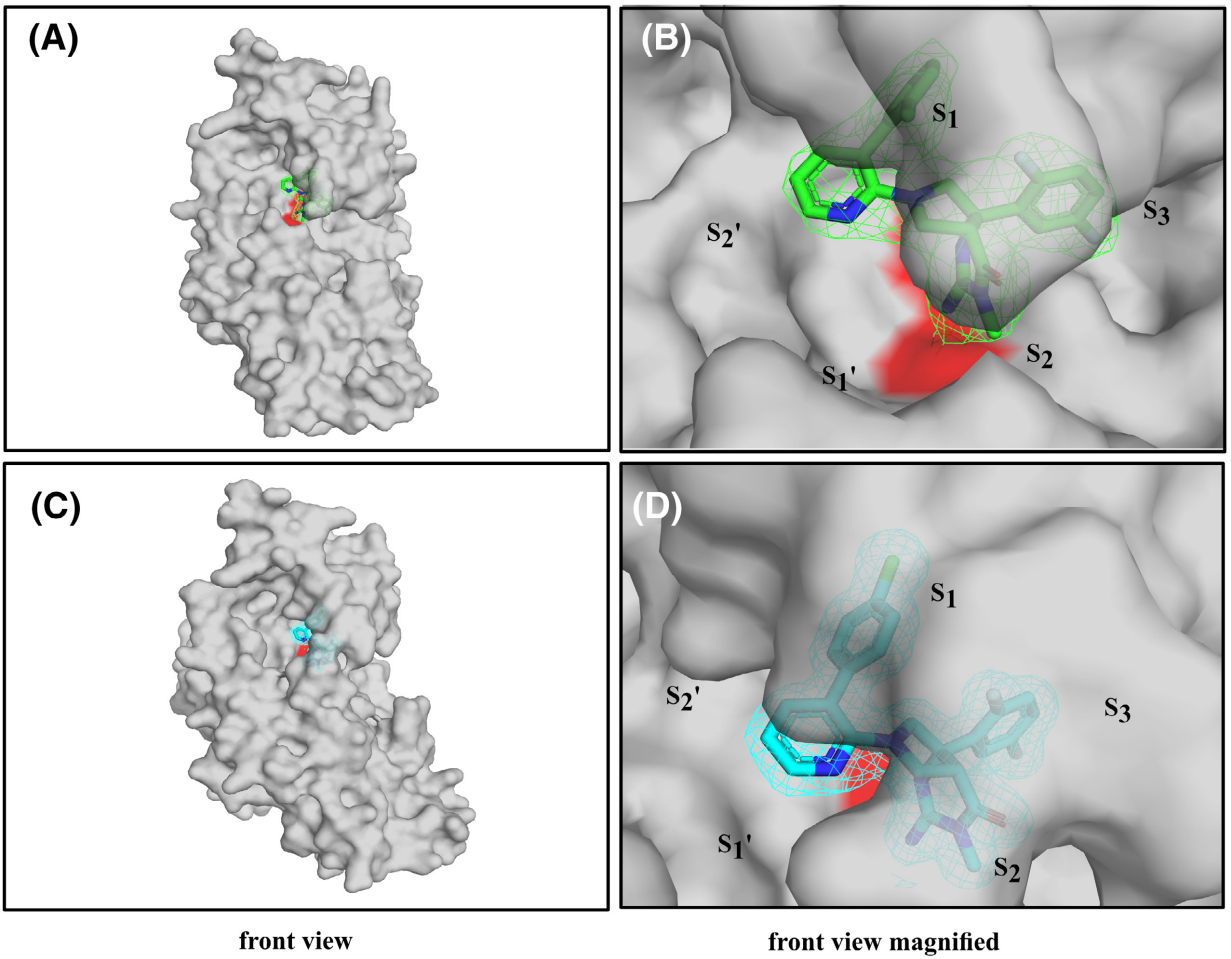
Structures of PvPMV–WM36 and PvPMV–WM48. (A) Electron density map (Omit) of WM36 (green) in complex with PvPMV (gray). (B) A magnified version of (A), the position of substrate binding pockets (S) shown, 2Fo‐2Fc density contoured at 1.0 σ with mesh representing electron density colored green. (C) Electron density map (Omit) of WM48 (cyan) in complex with PvPMV (gray), (D) A magnified version of (C), 2Fo‐2Fc density contoured at 1.0 σ with mesh representing electron density colored cyan. The location of the two active site aspartyl residues (D80 and D313) in the structure of PvPMV are represented by red coloring in each surface representation. The location of the two active site aspartyl residues, D80 and D313, in the structure of PvPMV are represented by red coloring in each surface representation. For all panels the PvPMV–WM36 and PvPMV–WM48 structures were determined by molecular replacement with the Autorickshaw server [44] using PvPMV‐WEHI‐842 structure (4ZL4.pdb). Further rounds of building and refinement with coot [45] and phenix [46] yielded the final model.

High‐resolution X‐ray crystallographic structures were obtained for PvPMV independently in complex with WM36 and WM48 at resolutions of 2.5 and 1.64 Å, respectively. Both molecules had an identical template except for the location of a chlorine moiety located in either the 2‐ (WM36) or 4‐ (WM48) positions on the terminal aryl group of the biphenyl moiety. The structures for the PvPMV–WM36 and WM48 complexes revealed that both molecules were aligned with the active site residues (D80 and D313) and positioned between the inner and outer (S_2_ flap) surfaces of the substrate binding pocket (Fig. 3A–D). The modeled structures for each compound fit comfortably within the bounds of each 2Fo‐Fc density contoured at 1.0 σ, represented by a mesh surface (Fig. 3B,D). An RMSD equal to 0.29 (332 to 332 atoms) shows their overall structures are very similar when overlaid. Their RMSD values for alignment to a structure with another PvPMV inhibitor complex, 4ZL4 (PDB), were also of similar value with the PvPMV–WM36 rmsd = 0.37 (312 to 312 atoms) and PvPMV–WM48 rmsd = 0.29 (310 to 310 atoms).

**Fig. 4:**
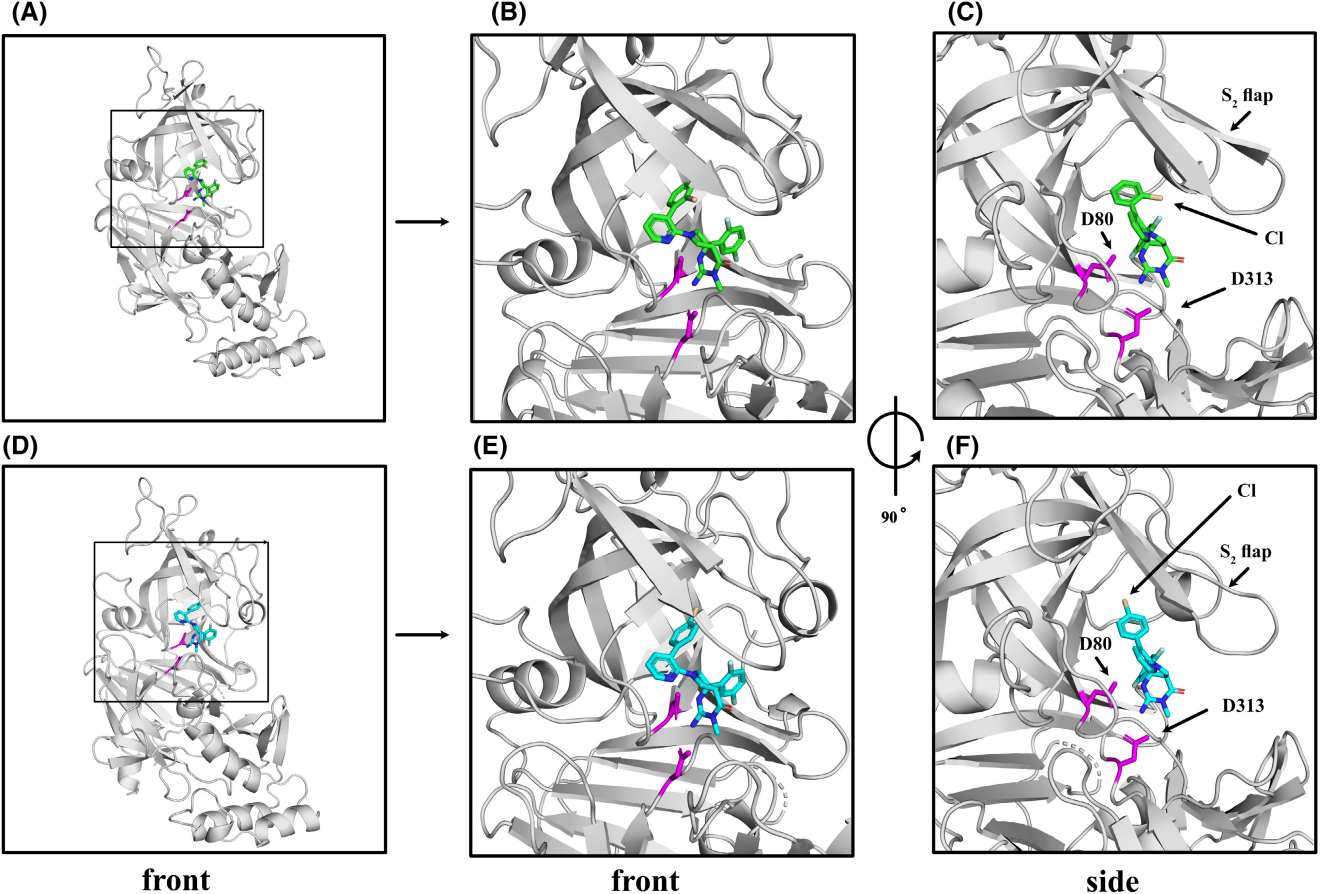
Graphical representation and comparison of the structures for PvPMV–WM36 and PvPMV–WM48. (A) Front view showing a cartoon of the entire PvPMV–WM36 complex. Box shows magnification area for (B) and (C). (B) Magnified front view with WM36 shown in green, active site aspartic acid residues (D80 and D313) highlighted in magenta, and PvPMV in gray. (C) Side view (90° anti clockwise rotation about the vertical axis of (B)) with the position of 2‐chlorine atom of the chlorobenzene moiety (Wheat in color) indicated by an arrow. Other structural features for orientation include the active site D80 and D313 (magenta) and the S_2_ Flap, which covers the front of the catalytic cleft. (D) Front view showing a cartoon of the entire PvPMV–WM48 complex. Box shows magnification area for (E) and (F). (E) Magnified front view with WM48 shown in cyan, active site aspartic acid residues (D80 and D313) highlighted in magenta, and PvPMV in gray. (F) Side view (90° anticlockwise rotation about the vertical axis of (E)) with the position of para chlorine atom of the chlorobenzene moiety (Wheat in color) indicated by an arrow. Other structural features for orientation include the active site D80 and D313 (magenta) and the S_2_ flap. For all panels the PvPMV–WM36 and PvPMV–WM48 structures were determined by molecular replacement with the Autorickshaw server [44] using PvPMV‐WEHI‐842 structure (4ZL4.pdb). Further rounds of building and refinement with coot [45] and phenix [46] yielded the final model.

WM36 and WM48 interact with PMV with a similar binding mode but subtle differences in interactions with the surfaces of the S_1_ and S_2_′ pockets lend to potency differences observed with these compounds. The imino‐pyrimidinone head group, for each, was orientated to enable interaction with the active site side chains of aspartic acid residues D80 and D313 at the S_1_ pocket of the catalytic domain (Fig. 4A–F). The positioning of the chloro‐substituted biphenyl moiety enabled interactions with the S_1_ and S_2_′ pockets of the substrate binding cleft. While the positioning of the chlorine in the 4‐position leads to increased interaction of WM48 with the roof of the S_1_ binding pocket, which was not observed in the PvPMV–WM36 structure. The difluoro aryl moiety at the other end of the inhibitor template spans the depth of catalytic cleft and participates in interactions in the S_1_ and S_2_ pockets (Fig. 4).

The S_2_ loop of PvPMV covers the front of the substrate binding cleft in the presence of bound inhibitors (4ZL4, 8TYF (PvPMV–WM36) and 8TYG (PvPMV–WM48)). However, in the structure for PvPMV–WM48 the electron density distribution (2Fo‐Fc) for residues Q136 to I145 indicates dual occupancy. This leads to a maximum deviation of 5 Å in the lower S_2_ loop position at residue E141 and indicates that this part of the S_2_ flap is mobile within the crystal structure (Fig. S1). The reorientation of the chlorine substitution from the ortho to para position on the aromatic ring results in decreased interaction of WM48 with the lower region of the loop and increased mobility in this region.

**Fig. 5:**
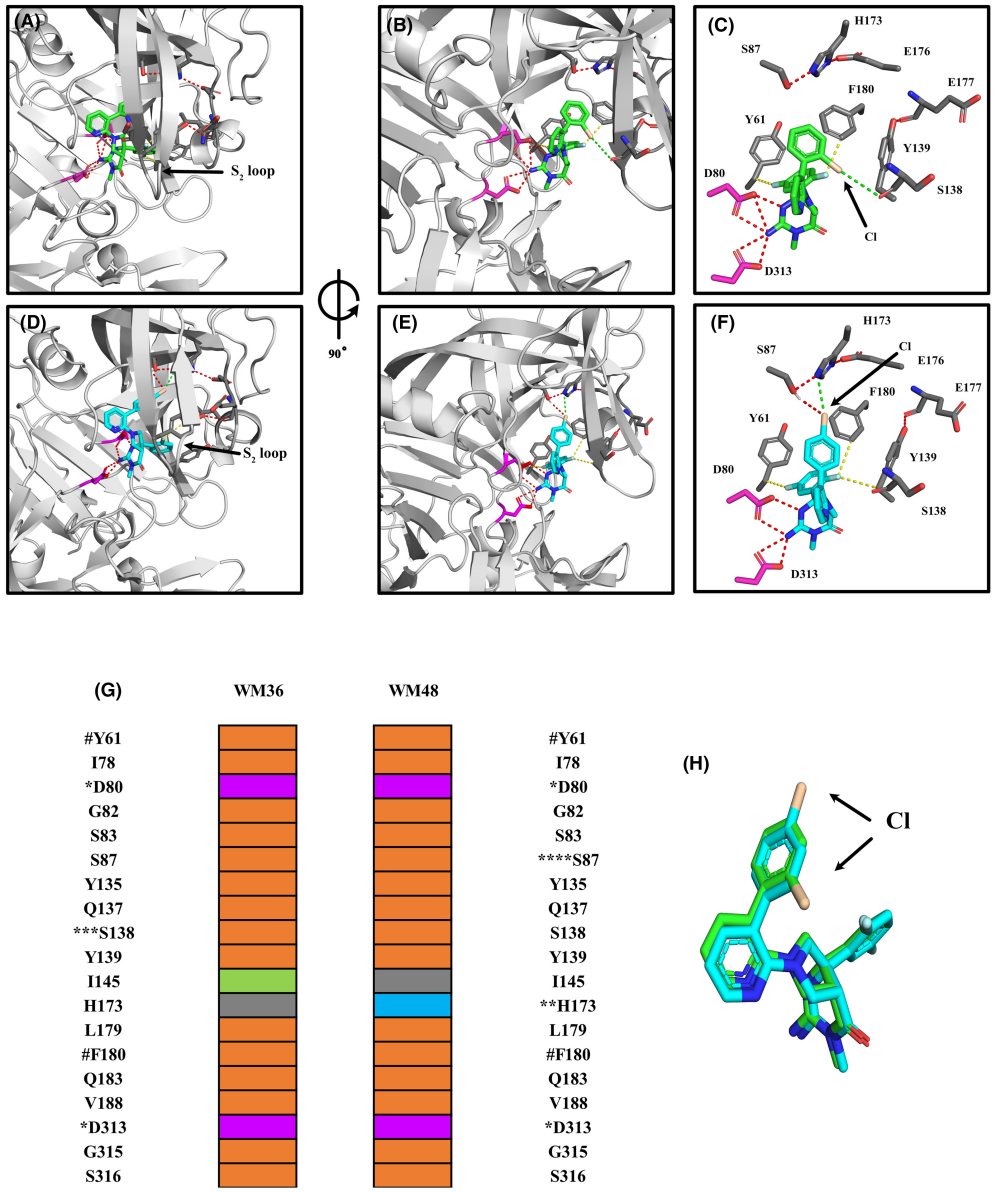
Surface interactions at the interface of inhibitor‐substrate for the structures of the PvPMV–WM36 and PvPMV–WM48 complexes. (A) and (B) shows front and side views (90° anti clockwise rotation about the vertical axis) of the hydrogen and halogen bonds occurring between WM36 (green) and the catalytic cleft of PvPMV (gray). (C) A simplified deconstructed side view (as for (B)) showing the hydrogen and halogen bonds occurring between WM36 and the catalytic cleft of PvPMV. Red dashes = conventional hydrogen bonds, green dashes = halogen bond and yellow dashes = F–H–C type hydrogen bonds. The chlorine atom (wheat in color) of chlorobenzene moiety (ortho position) is indicated by an arrow. (D) and (E) show front and side views (as per (A) and (B)) of the hydrogen and halogen bonds occurring between WM48 (cyan in color) and the catalytic cleft of PvPMV (gray). (F) A simplified deconstructed side view (as per (C)) showing the hydrogen and halogen bonds occurring between WM48 and the catalytic cleft of PvPMV. The Cl atom of chlorobenzene moiety (para position) is colored wheat and indicated by an arrow. E176 does not interact with either inhibitor but is shown due to its hydrogen bond to H173, which contributes to the stability of the catalytic cleft roof in this area. (G) Van der Waals surface interactions (< 4 Å) between PvPMV and WM36 and WM48, interactive residues are color‐coded as orange = residues that interact with either inhibitor, magenta = location of active site aspartic acid residue and interacts with both inhibitors, lime = residues that interact specifically with WM36, cyan residues that interact specifically with WM48 and gray = residue does not interact with specific inhibitor. *Involved in active site hydrogen bonding in both structures, **Involved in a halogen bond between 4‐chlorine atom and WM48, ***Involved in a halogen bond between 2‐chlorine atom of WM36, ****Involved in hydrogen bonds with H173 and the 4‐chlorine atom of WM48, ^#^Involved in F–H–C type hydrogen bonds in WM38 and WM48. Interactions between PvPMV and WM48 shown in the table are those based on those found for S2 loop conformer A described in Fig. S1. (H) Models for the WM36 (green) and WM48 (cyan) were obtained from the aligned structures for PvPMV–WM36 and PvPMV–WM48 with the chlorine atom wheat in color. For all panels the PvPMV–WM36 and PvPMV–WM48 structures were determined by molecular replacement with the Autorickshaw server [44] using PvPMV‐WEHI‐842 structure (4ZL4.pdb). Further rounds of building and refinement with coot [45] and phenix [46] yielded the final model.

WM36 and WM48 have a common core structure and differ only by positioning of the chlorine atom located in the pendent aryl moiety. Even though such changes in the structure of the moiety are subtle, they can still result in significant changes in the interactive surface and consequently the affinity for each compound for PMV. Within the PvPMV–WM36 complex hydrogen bonds of interest occur between S87, H173, and E176 in the roof of the S_1_ pocket and Y139 (S_2_ loop) and E177 in the S_2_ pocket (Fig. 5A–C). The later hydrogen bond help stabilize the Y139 of the *S*_*2*_ loop that was pushed aside to make room for WM36 to bind to PvPMV. The fluorine atoms of the difluoro aryl moiety of WM36 are orientated such that they can participate in F–H–C type hydrogen bonds with F180 aromatic H–C and H–C of the CB carbon of the Y61 side chain (Fig. 5A–C). Orientation of the chlorine atom from the pendent aryl moiety (ortho position) indicates the existence of a weak halogen bond (3.8 Å) between the electropositive sigma hole of the chlorine atom and the main chain carbonyl of S138 (Fig. 5A–C). This interaction appears to be important for additional stabilization of the S_2_ loop compared with the S_2_ loop stability in the WM48 structure.

The shift in location of the chlorine from the chlorobenzene moiety to the para position in WM48 resulted in an increased stabilization of this compound in the roof of the S_1_ pocket of the substrate binding cleft for PvPMV. This occurs via a unique property of the halide atoms, which enables the chlorine to act as both a Lewis acid and Lewis base [37, 38]. The electropositive sigma hole on the chlorine atom is electron poor and enables the formation of a halogen bond (3.2 Å) with the nitrogen (NE2) of H173 side chain. A hydrogen bond occurs between the chlorine atom and the hydroxy hydrogen of S187 and was perpendicular to the axis for the halogen bond. This allows WM48 to be anchored, via the halide interactions with S187 and H173, into a highly stabilized region of the S_1_ pocket roof (Fig. 5D–F). The shift of the chlorine atom from the ortho to para position has also resulted in decreased interaction and consequently increased mobility in the lower section of the S_2_ loop (residues Y139 to Q144) (Figs 4 and 5, Fig. S1). This was reflected in the distribution of the electron density about this region and the requirement to fit these residues of the model into dual occupancies.

The Van der Waal surface interactions (< 4 Å) that occur between the PvPMV–WM36 and PvPMV–WM48 complexes are summarized in Fig. 5G. Not unexpectantly, the interactive surface for each inhibitor with PvPMV are very similar in the catalytic cleft region (Fig. 5H). The major difference between these two interactive surfaces results from the rearrangement of the chlorine atom to the para position on the aromatic ring for WM48. This enables enhanced tethering of WM48 to the S_1_ pocket roof via a unique interaction with H172 via a halogen bond and an increased interaction with S87 via a hydrogen bond (Fig. 5, Fig. S2). The differences observed for I145 result from minor differences in the orientation of this residue, which appears to be influenced by the positioning of the substituted chlorobenzyl moiety in each structure.

**Fig. 6:**
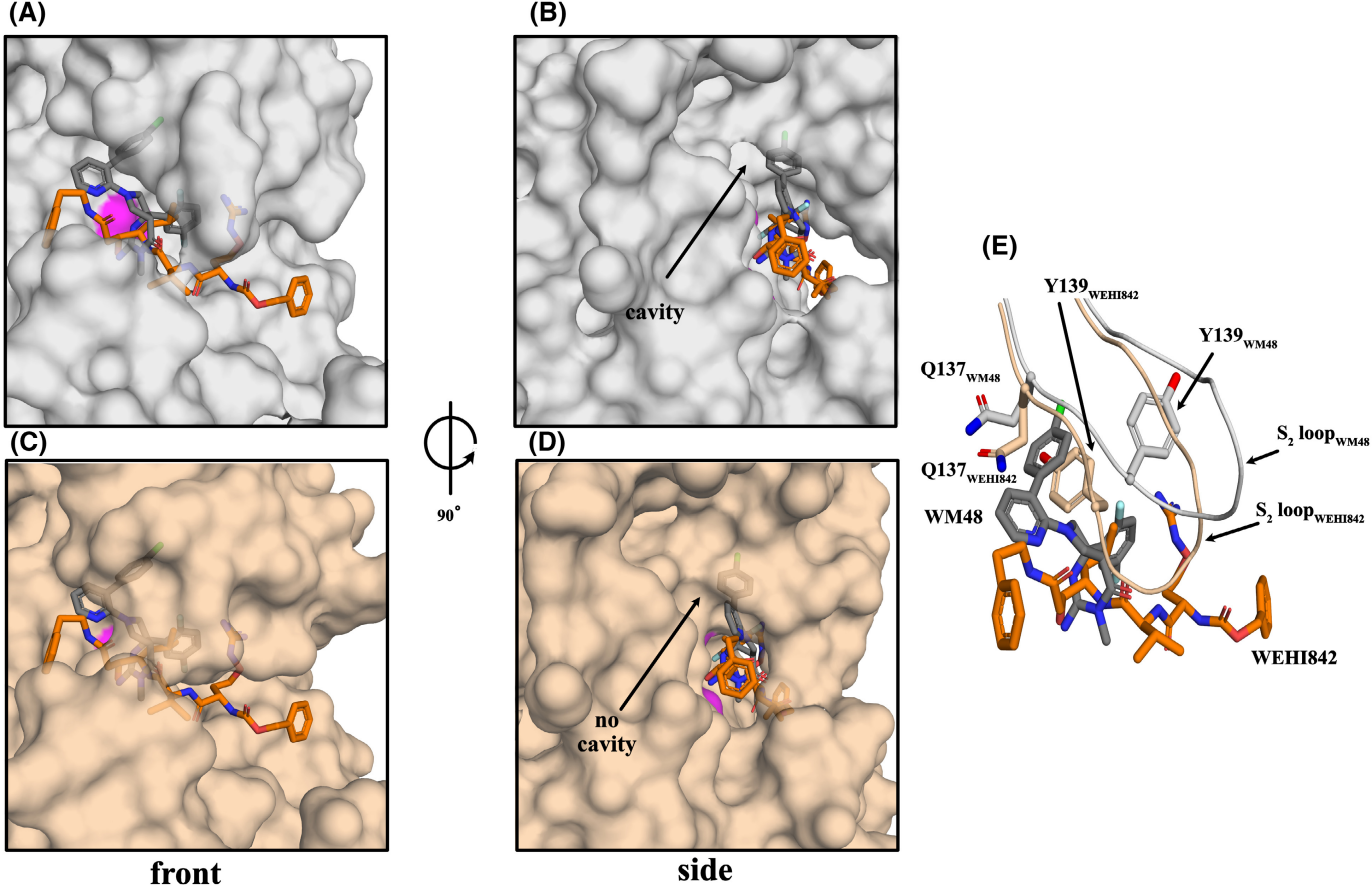
Comparison of inhibitor positioning and inhibitor induced topographies within the catalytic cleft of PvPMV. (A) Surface representation for the PvPMV–WM48 structure (gray) with the aligned model for peptidomimetic compound WEHI‐842 (orange). The aligned structures for 8TYG and 4ZL4 have an RMSD = 0.29 Å (310 to 310 atoms). (B) A side view of (A) obtained by a 90° anticlockwise rotation about the vertical axis. (C) Surface representation of the structure for PvPMV‐WEHI‐842 (4ZL4, orange) with the aligned model for the small molecule inhibitor, WM48 (8TYG, gray). (D) A side view of (C) obtained by a 90° anticlockwise rotation about the vertical axis. Arrow indicates the presence of a roof cavity at the interface of the S_2_′/S_1_ pockets in the 8TYG structure for PvPMV–WM48 (B) and the absence of the cavity in the 4ZL4 structure for PvPMV‐WEHI‐842 (D). (E) A cartoon overlay of the structures for the S2 loop of PvPMV found in complex with WM48 and WEHI‐842. Orientation of the loop and in particular residues Q137 and Y139 is governed by inhibitor contacts. These residues (gray) are positioned to make space for and accommodate the binding of WM48 resulting in the formation of a cavity which encapsulates the 4‐chlorobenzene moiety (see also (B)). In the absence of WM48 these same residues are orientated inwards toward the cavity space, as seen in the PvPMV‐WEHI842 structure which has no inhibitor contacts in this region of the S2 loop (wheat). The cavity does not form in this conformation of the S2 loop (see also (D). For relevant panels PvPMV–WM36 and PvPMV–WM48 were determined by molecular replacement with the Autorickshaw server [44] using PvPMV‐WEHI‐842 structure, 4ZL4.pdb). Further rounds of building and refinement with coot [45] and phenix [46] yielded the final model.

The relative positioning in the PvPMV catalytic cleft of the drug‐like WM48 relative to the peptidomimetic inhibitor, WEHI‐842 (PDB: 4ZL4), was different yet both compounds significantly inhibited the activity of PvPMV (Fig. 6) [29, 35]. Although the IP head group of WM48 and the hydroxy moiety of the statine (a modified amino acid that is an aspartyl protease inhibitor [28]) in WEHI‐842 both interact with the active site aspartyl residues (D80 and D313) of PvPMV, their overall interactive surfaces are different. The WM48 molecule binds closer to the roof of catalytic cleft for PvPMV (S_2_′, S_1_′, and S_1_). While WEHI‐842, a PEXEL substrate mimic, binds relatively lower in the cleft and has interactions mainly involving the S_1_ to S_3_ pockets, which define the natural substrate specificity of PMV (Fig. 6). Inhibitors can also induce changes in the interactive surface at their binding site. For example, the chlorobenzene moiety of WM48 is found to be located within a roof cavity found at the interface of the S_2_′/S_1_ pockets (Fig. 6B). This cavity is not found in the PvPMV WEHI‐842 structure (Fig. 6D). A comparison of the two structures reveals that the orientation of the S_2_ loop and in particular two residues (Q137 and Y139) determines whether a cavity can form to accommodate the 4‐chlorobenzene moiety of WM48. In this structure, Q137 and Y139 are positioned to enable WM48 to bind (Fig. S2). Residue Y139 is pushed up against the inner surface of the S_2_ loop, while the loop itself also moves sideways and twists outward resulting in the formation of a cavity in the S_2_′/S_1_ roof surface (Fig. 6B,E). Increased interactions between WEHI842 and the lower region of the S_2_ loop (PDB: 4ZL4) make this area less mobile than observed in the PvPMV–WM48 structure (PDB: 8TYG). When no cavity was observed, as in the PvPMV–WEHI842 structure, both Q137 and Y139 are orientated inwards toward the central space of the catalytic cleft where they do not sterically interfere with the positioning of WEHI842. However, in this S_2_ loop conformation, Q137 and Y139 would collide with WM48. In PMX structures (PDB: 7TBD, 7TBE and 8TYH), F276 is also pushed against the inner surface of the S_2_ loop to enable WM4 and WM382 to bind. But in this instance, no cavity formation is required to accommodate the compounds. The S_2_ loop remains in position as the residues at the lower hairpin region of the loop engage the compounds with the conformation of the loop being similar to that found in the apo structure of PvPMX (PDB: 8TYH) [22]. Hence, the molecular structure of both inhibitors plays a significant role in the “sculpturing” of the interactive surface within the catalytic cleft and reveals inhibition of PMV can be achieved by targeting different regions within the catalytic cleft. Some of these areas may also overlap with those shared by other inhibitory compounds that target‐related enzymes.

**Fig. 7:**
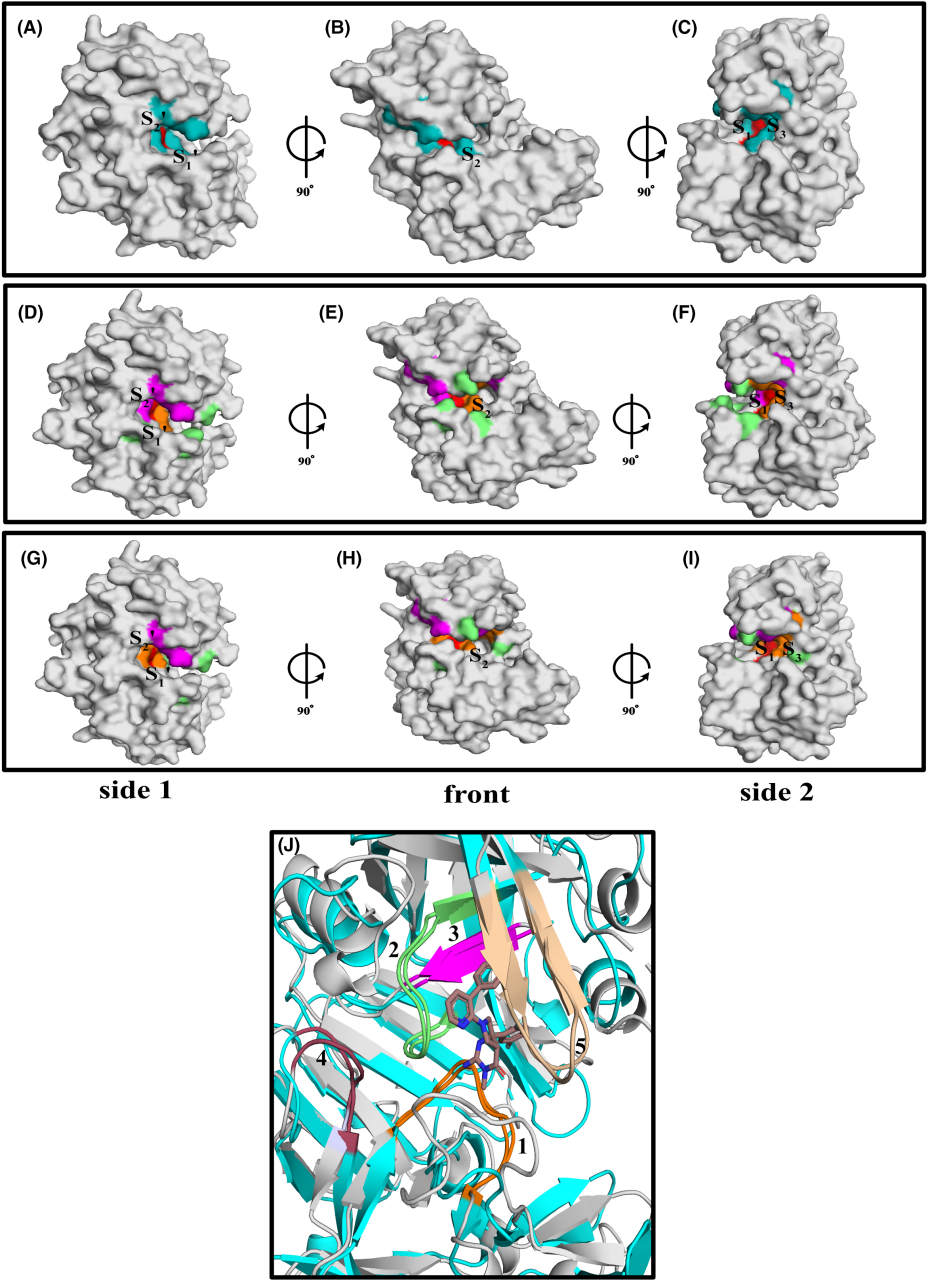
Comparison of the interactive sites for inhibitors of PvPMX. (A–C) The location of residues involved in surface interactions (< 4 Å) in the PvPMV–WM48 complex transposed onto structurally equivalent locations in the aligned structure for PvPMX apo. (A) Side view 1 (taken from the S′ side of the cleft) of the surface schematic for PvPMX apo structure (gray, PDB:8TYH). Cyan = residues from the PvPMV–WM48 surface interactions (< 4 Å) transposed onto PvPMX apo. Red = location of the active site aspartic acid residues and interact with inhibitor. (B) front view resulting from a 90° clockwise rotation about the vertical axis. (C) Side view 2 (taken from the S side of the cleft) resulting from a 90° clockwise rotation about the vertical axis of the front view (B). (D–I) Comparison of the location for the PvPMV–WM48 surface interactions (< 4 Å) locations transposed onto PvPMX apo (Fig. 5A–C) to the location of residues determined to interact with potent inhibitory compounds of plasmepsin X. (D) Side view 1 (taken from the S′ side of the cleft), surface representation of PvPMX apo comparing the interactive residues involved with binding WM48 and WM4 (PDB:7TBE). Magenta = WM48‐specific interactions, orange = WM48 and WM4 common interactions (red = common, active site aspartic residues) and lime = WM4‐specific interactions. (E) front view resulting in a 90° clockwise rotation about the vertical axis of (D). (F) Side view 2 (taken from the S side of the cleft) resulting from a 90° rotation about the vertical axis of (E). Positions of relevant substrate binding pockets are shown by S. (G) Side view 1 (taken from the S′ side of the cleft), surface representation of PvPMX apo comparing the interactive residues involved with binding WM48 and WM382 (PDB: 7TBD). Magenta = WM48‐specific interactions, orange = WM48 and WM382 common interactions and lime = WM382‐specific interactions. (H) front view resulting in a 90° clockwise rotation about the vertical axis of (G). (I) Side view 2 (taken from the S side of the cleft) resulting from a 90° rotation about the vertical axis of (H). The positions of relevant substrate binding pockets are shown by S or S′. (J) Structurally conserved regions in the aligned carbon main chain structures for PvPMX (PDB: 8TYH) and PvPMV (PDB: 8TYG). Five regions were identified in the aligned structures that were < 7 Å from the WM48 inhibitor. Four of these regions had clusters of main chain carboxamides and/or side chains with the potential to participate in hydrogen bonding. The fifth region contains the S_2_ loop, although more variable in structure than regions 1–3, the benefits in engaging binding to this loop are known to significantly increase inhibitor affinity [22]. Region 1 = orange color, contains second active site Asp. Sequence for PvPMX = F420‐N427 and equivalent region in PvPMV = V312‐T319. Region 2 = green color, contains the first active site Asp. Sequence for PvPMX = T232‐W238 and equivalent region in PvPMV = T81‐S87. Region 3 = magenta color, located in the roof of the S_1_ pocket. Sequence for PvPMX = T232‐W238 and equivalent region in PvPMV = T81‐S87. Region 4 = raspberry color located near the roof of the S_2_′ pocket. Sequence for PvPMX = E393‐Y396 and equivalent region in PvPMV=Y285‐Y288. Region 5 = wheat, S_2_ loop. Sequence for PvPMX = H273‐I281 and equivalent region in PvPMV = M136‐S146. Gray cartoon schematic represents the structure for PvPMX apo and the cyan cartoon schematic is for the PvPMV–WM48 complex with WM48 carbon atoms colored brown, nitrogen atoms blue and oxygen atoms red. For relevant panels the PvPMX apo structure was determined by molecular replacement with the Autorickshaw server [44] using PvPMX‐WM382 structure (7TBD.pdb) as the search model. PvPMV–WM36 and PvPMV–WM48 were determined by molecular replacement with the Autorickshaw server [44] using PvPMV‐WEHI‐842 structure (4ZL4.pdb). Further rounds of building and refinement with coot [45] and phenix [46] yielded the final model.

PMV and PMX have very different functions in the parasite, yet their aligned structures around the area of the catalytic cleft have an RMSD = 1.751 Å (Cα 125 atoms of 125 atoms). This indicates the potential for the discovery of compounds that have inhibitory properties against both enzymes. WM4 is an inhibitor of PMX, and the specificity for its inhibitory properties was due to interactions with residues in the S_2_′, S_1_′, and S_1_ pockets of the catalytic cleft [18]. WM48 has been found to have moderate inhibitory activities against both PMV and PMX in biochemical assays and has interactions in the S_2_′ and S_1_ pockets potentially providing a common interactive area that would enable dual inhibition of these two enzymes, using a single compound. Subtle structural differences in IPF analogs enhance activity in conjunction with the identification of areas of structural similarity (Tables 1, 2, 3). A surface representation of PvPMX enzyme domain apo structure (PDB: 8TYH) shows the structurally equivalent residues labeled (colored cyan) that correspond with WM48 interactions (< 4 Å) observed in the aligned *Pv*PMV–WM48 complex (PDB: 8TYG) (Fig. 7A–C). The structurally equivalent residues identified in PvPMX have a similar distribution to those observed in the 8TYG structure with a significant presence in the S_1_′ and S_2_′ pockets of the catalytic cleft.

The surface representation for the PvPMX apo structure shows the residues that interact (< 4 Å) with WM4 (PDB:7TBE) and those structurally equivalent to the surface interactions between WM48 and PvPMV (Fig. 7D–F). Although WM4 and WM48 have PMX inhibitory properties, the positions in the catalytic cleft for the residues they interact with in the S_1_′ and S_2_′ pockets are not the same. This was not surprising as both inhibitors are derived from different core molecular templates that will determine their relative binding sites within the catalytic cleft. It also indicates in addition to areas of common interaction shared by both inhibitors in the S_1_ and S_2_ pockets (Fig. 7C–E, orange color), there was also a significant number of residues in the S_1_′ and S_2_′ pockets that can potentially be targeted to enhance dual inhibition of PMV and PMX. Affinities toward both enzymes could be increased by targeting regions with identical or similar residues.

The surface representation for the PvPMX apo structure showed the residues that interact (< 4 Å) with WM382 (dual PMIX/X inhibitor, PDB:7TBD) and those structurally equivalent to the surface interactions between WM48 and PvPMV. WM382 shares areas of common interaction with WM48 largely in the S_1_ and S_2_ pockets (orange) (Fig. 7G–I). However, the specific interactions for each inhibitor lie at opposite ends of the catalytic cleft. WM48‐specific interactions (magenta) are focused in the S_1_′ and S_2_′ pockets while those specific for WM382 (lime) are found in the S_2_ and S_3_ pockets. The structural topography within the S′ pockets for PMIX are thought to differ to that for PMX, hence the lack of interactions between WM382 and PMX in this area [18].

A cartoon schematic displaying the overlaid main chain carbon structures for PvPMV–WM48 and PvPMX apo shows the regions that are structurally similar (Fig. 7J). Both PvPMV–WM48 and PvPMX apo structures have a residue side chain and/or main chain carboxamide groups within < 7 Å of the WM48 inhibitor that could be targeted for improving inhibitor affinity. Sites 1–2 (orange and lime), in both enzymes, surround the active site Asp residues and contain side chain groups and main chain carboxamides that can be targeted for hydrogen bonds. Site 3 (magenta) contains hydrophobic side chain groups and main chain carboxamides, and Site 4 (brown) is in an area that has a cluster of hydrophilic residues, observed in both PMX and PMV, which could be targeted for additional hydrogen bonding interactions. Site 5 (wheat) is the S_2_ loop and although the sequences in this site are not overly similar, side chain groups and main chain carboxamides are potentially available for hydrogen bonding that could add extra stability to the enzyme–inhibitor complex through attachment to not only the back wall but also the front wall of the catalytic cleft.

### Modeling the IPF analogs into the structure for PfPMX

**Fig. 8:**
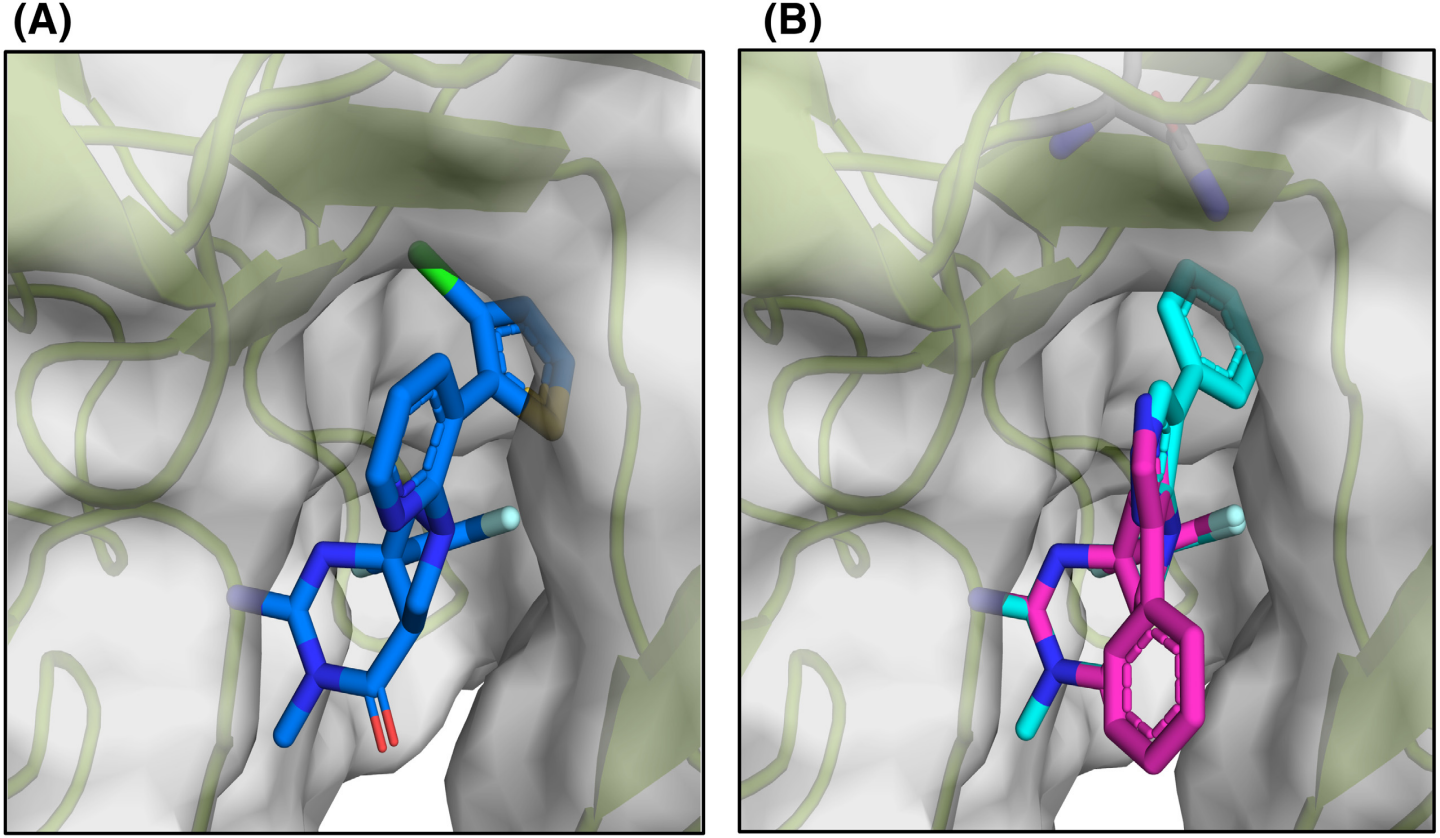
Modeling of thiophene and pyrazole analogs WM396 and WM447 with PfPMX. (A) thiophene analog WM396 (blue) with surface and cartoon representation of PfPMX. (B) Pyrazole analog WM447 binding mode 1 (aqua) and binding mode 2 (magenta). Pyrazole N–H interacting Asn 271 is shown at the top of the panel. clc drug discovery workbench software (version 2.5.1) was used to model analogs WM396 and WM447 to the X‐ray structure of PfPMX (PDB 7TBC) as described previously [18]. The structure of PvPMV–WM48 was aligned and overlaid with PfPMX providing a template for modeling and positioning the IPF scaffold.

Using the X‐ray structures of WM36 and WM48 in complex with PMV, and WM382 in complex with PfPMX (PDB: 7TBC) [18] as small molecule and protein templates, we modeled the thiophene and pyrazole analogs WM396 and WM447 in complex with PfPMX (PDB: 7TBC) to understand the structural basis for their PMX inhibition. Modeling showed that the thiophene ring was suitably accommodated in the S_1_ pocket of PMX (Fig. 8A), while the phenyl ring (e.g. analog **24**) appeared to clash with electron density in the roof of the S_1_ pocket of PMX (not shown), supporting the observation that the thiophene system (analogs **24**–**26**) inhibited PMX (IC_50_ values < 0.070 μm) while phenyl or substituted phenyl derivatives showed modest inhibition of PMX. The 3‐chloro substituent appeared to fix the thiophene ring in a particular orientation in the S_1_ pocket of PMX, providing supportive structural evidence that this derivative (compound WM396) was interacting more potently (×8) against PMX (IC_50_ 0.009 μm) than the unsubstituted comparator analog **24**.

Modeling of the pyrazole analog WM447 with PfPMX (PDB: 7TBC) was not as definitive as the modeling of the thiophene analog, as there were two potential binding modes (Fig. 8B). The first binding mode projected the P_1_ terminal phenyl ring in the same orientation as WM36 and WM48 in complex and drives the phenyl ring deeper into the S_1_ pocket giving a steric clash with electron density at the roof of the PMX S_1_ pocket. The second binding mode does not engage the S_1_ pocket of PMX, but instead, the orientation of the aryl ring is flipped 180°, outward and away from the catalytic cleft surface. In this pose, there was also potential for the pyrazole NH to engage with the side chain of Asn127 located at the periphery of the S_2_′ pocket. While the modeling does not give definitive evidence it illustrated potential binding modes and a rationale for the potent PMX activity exhibited by the pyrazole analog WM447 having potent PMX activity.

## Discussion

The aspartic proteases PMIX and PMX in *P. falciparum* are crucial targets for developing antimalarial drugs with a novel mode of action and a high barrier to resistance. This focus led to the discovery of WM382, a lead compound, and MK‐7602, a clinical candidate. Both are potent dual inhibitors of PMIX and PMX, with MK‐7602 advancing to Phase 1 clinical trials in humans. The closely related aspartic protease PMV is also a promising drug target, with potent peptidomimetic inhibitors developed against both the protease and parasite growth. The success in creating dual inhibitors of PMIX and PMX has opened the possibility of developing small molecules capable of inhibiting various combinations of PMIX, PMX, and PMV in *P. falciparum*. A compound library screen against PvPMV produced a hit compound WM36, which is comprised of an IPF scaffold. In this study, we explored the potential of IPF scaffold variants as inhibitors of the three proteases. This investigation resulted in the identification of *S*‐WM48, a compound that potently inhibits the *in vitro* protease activity of PMV, PMIX, and PMX. While the potency of *S*‐WM48 against PMV, PMIX, and PMX protease activity *in vitro* was encouraging the activity against *P. falciparum* requires considerable improvement highlighting the need for additional structural and biological studies to increase efficacy against the parasite.

WM36 demonstrates moderate inhibitory activities against PMV, PMIX, and PMX *in vitro*. A series of substitutions to the WM36 template has provided a foundation for further development of this compound class to target PMV and PMX, both of which are essential for parasite survival. The WM36 analogs, similar to the PMX‐specific inhibitor WM4 [18], interact with amino acids in the S_1_, S_1_′, and S_2_′ pockets of the catalytic cleft, defining their specificity. While these inhibitors share overlapping interaction regions in the S_1_ and S_2_ pockets, differences in template designs result in unique interactions within the S_1_, S_1_′, and S_2_′ pockets (Fig. 7C–E). These distinct binding interactions suggest potential for targeting additional residues in these pockets to achieve potent dual inhibition of PMV and PMX. WM382 [18], a dual inhibitor of PMIX/X based on the WM4 template, shares common interaction areas with WM48 in the S_1_ and S_2_ pockets of PMX when compared (Fig. 7G–I). However, their specific interactions occur at opposite ends of the catalytic cleft, indicating different regions promote binding of the PMIX/PMX dual inhibitor.

Based on these observations, further modifications to the biphenyl moiety of the WM36 family of inhibitors were explored to enhance their dual PMV/PMX inhibitory characteristics. The biphenyl moiety in the WM36 scaffold is positioned near the roof of the S_2_′ and S_1_ pockets in both PMX and PMV, an area where these enzymes appear to share similar structural features. We conducted numerous substitutions of the biphenyl moiety to determine the requirements for improved inhibitor binding. To understand the resulting modes of interaction, we analyzed structures of similar PMV and PMX inhibitor complexes.

The *S*‐isomers of both WM36 and WM48 have been crystallized in complex with PMV (Fig. 3 and Table S1, PDB: 8TYF and 8TYG). Although there were no significant changes observed in the surface topography of the S1 roof cavity in both aligned structures (Fig. S2) the increase in binding affinity for WM48 is due to the formation of a halogen and hydrogen bonds. Alignment of the two structures shows they have a similar binding mode (RMSD = 0.29 Å Cα 332 to 332 atoms; Figs 4 and 5). The subtle change from 2‐chloro to 4‐chloro on the terminal aryl ring of the biphenyl moiety leads to a significant increase in the binding affinity of WM48. The increases in binding affinity for WM48 is due to the formation of a halogen bond with H173. The carbon–chlorine bond is also proximal to the hydroxy hydrogen of S87 enabling the formation of a hydrogen bond with the electron orbitals of the chlorine atom and occurs perpendicular to the axis of the halide bond. These increased interactions with the substituted 4‐chloro enable WM48 to be anchored more tightly into the roof cavity in the S_1_ pocket (Fig. 5). This same cavity is not evident in the structure for PvPMV in complex with the peptidomimetic inhibitor WEHI‐842 (Fig. 6, PDB: 4ZL4) [29] as the interactive site for this inhibitor is located much lower in the catalytic cleft than for WM48. This indicates that inhibition of PMV can be achieved by manipulating the structure of inhibitors to target different regions and the surface topographies within the catalytic cleft of PMV and that different scaffolds of these compounds can lead to differences in the conformation resulting from the induced fit with PMV. However, understanding the conformational changes induced in the enzyme structure by compound binding and then how these changes can be further used to enhance the desired outcome via compound design leading to improved affinities toward changes in the molecular surface within the catalytic cleft, still relies heavily on determination of structures for these enzyme–inhibitor complexes.

A similarly positioned surface cavity in the roof of the S_1_ pocket of the catalytic cleft for PMX is also observed in several PMX apo and PMX/inhibitor structures (PDB: 7TBB, 8TYH, 7TBC, 7TBD, 7TBE) (Fig. S3A–D). The presence in both apo and inhibitor structures suggests this cavity is present without the influence of an inhibitor. Alignment of PvPMX apo structure (PDB:8TYH) with the structure for PvPMV–WM48 (PDB: 8TYG, Fig. 7) and identifying those residues in PMX that are equivalent to those of PvPMV that are interacting with WM48 reveals that H173 (PvPMX V305) and S87 (PvPMX W238) equivalents are not found in the PMX sequence at the same locations and halogen and hydrogen bonds that anchor the WM48 inhibitor to the roof of the S_1_ pocket cannot be formed by the replacement of these residues. Instead, V305 is orientated to contribute to the hydrophobicity of the roof for the S_2_ pocket. While W238 along with I274, I281, and I327, form the edge of the S_1_ pocket located on the roof of the cleft in PMX. These residue changes lead to a smaller and more restrictive S_1_ pocket in PMX (Fig. S3). The terminal 4‐chloro aryl ring of the biphenyl moiety is too large for this pocket and would clash with the S_1_ pocket wall. Although modest improvement in the affinity of WM48 for PMX is observed the induced fit to this area is not optimal. WM36 does not fit into this same pocket as well as WM48, due to the bulky 2‐chloro aryl ring substitution, which projects outwards from the catalytic cleft. Like WM48, WM36 is too large to fit comfortably within the PMX S_1_ pocket (Figs S2 and S3E–H).

The 3‐chloro‐substituted analog WM396 provided eightfold better inhibition of PMX compared with the other two thiophene‐derived analogs, and this appears to be due to the ability of the chlorine atom to participate in halogen and hydrogen bond interaction with the main chain oxygen of PfPMX N271, and the hydroxy moiety for PfPMX S269 (Fig. S4A,C,E,G). Alignment with the PvPMV–WM48 structure reveals that the potential for a halogen and hydrogen bond also exists in the equivalent area of the S_1_ pocket of PMV via interactions with PvPMV S85 (halogen bond) and PvPMV S83 (hydrogen bond). However, the pocket in PMV is much larger than in PMX (Fig. S4B,D,F,H), which prevents compound WM396 (Table 2) from tightly binding to PvPMV. It is indicative that the difference in S_1_ pocket sizes is a key driver of selectivity between PMX and PMV.

The thiazole and pyrazole rings were used as bioisosteric replacements for the pyridyl ring, and of these, compound WM447 was found to have the highest inhibitory effects against PMX (IC_50_ 0.064 μm) while only moderately inhibiting PMV (IC_50_ 0.683 μm) (Table 3). To investigate the mode of interaction, WM447 was modeled into the structure of PfPMX (PDB: 7TBC). Modeling predicted two binding modes arising from rotation of the biaryl moiety around the carbon nitrogen bond of the pyrrolidine nitrogen. In one rotameric binding mode (binding mode 1, Fig. 8B), the pendant aryl ring enters the S_1_ whereby the phenyl ring tightly abuts amino acids on the S_1_ roof cavity and is skewed away from the orientation of the equivalent phenyl ring observed in WM48 in the structure 8TYG. This potentially leads to steric clashes with residues in this region of the roof pocket in both PMX and PMV. Moreover, the nitrogens on the pyrazole are predicted not to form any interactions in either plasmepsin X or V. In the alternative rotameric binding mode (binding mode 2, Fig. 8B), the interactive N atoms of the pyrazole ring do not substantially enter the cavity of the S_1_ pocket instead they are orientated to engage residues 3–4 Å further toward the surface exposed area at the S_2_′/S_1_ boundary (Fig. S5). The proximity of the heterocyclic 1‐N and 2‐N to the side chain amide of PfPMX N271 (ND2) and side chain hydroxy of S269, respectively, indicates the potential for hydrogen bonding to occur (3.5 and 3.7 Å, Fig. S7G) and was reflective of the PMX inhibition observed. For PvPMV, the 1‐N of the heterocyclic ring is orientated toward the S85 side chain and S84 main chain amide with potential to form a hydrogen bond (Fig. S7H). However, importantly, there is a lack of a suitable surface in this region of the substrate binding cleft, to assist with orientation and enhancement of compound binding to residues in both PMX and PMV (Fig. S7A–F).

The systematic changes in the original WM36 biaryl moiety have moved the interactive surface for the IPF template progressively across the roof of the S_1_ and into adjoining S_2_′ pocket. This has resulted in the identification of a conserved cavity in the S_2_′ pocket, which contains a cluster of hydrophilic residues in PMX and PMV that are approximately 7 Å from WM447 (rotomer 2) with the potential for targeting by this class of compound (Fig. S6). Changes in the position and substitution of the phenyl ring (green) and utilization of the 5‐position of the pyrazole ring (magenta) would be a strategy to enhance engagement and affinity toward this cluster of residues.

The structural analysis of the binding sites for positive hits generated by the compound screen has uncovered some binding subsites that translate into large increases in the potency for PMX. Although similar increased potency changes toward PMV were not observed, a better understanding of inhibitor/plasmepsin structures within these binding sites was determined and can assist with further medicinal chemistry discovery strategies to improve compound potencies against PMX and PMV.

## Materials and methods

### Plasmepsin V, IX, and X fluorogenic assays

These fluorogenic assays follow protocols previously described [14, 18, 39, 40]. Compound potency was assessed with 10 point, threefold dilution series starting at 100 μm in duplicate per experiment and repeated in three independent experiments. Data were normalized to percent inhibition relative to 1% DMSO (high control) and either 1 μm WEHI‐600 for PMV and 1 μm WM382 for PMIX/PMX (low controls). IC_50_ values (relative inflection of dose–response curve) were calculated using a nonlinear regression four‐parameter fit analysis in dotmatics 5.3 (Dotmatics, Boston, MA, USA) and spotfire 7.11.1 (Goteborg, Sweden) software. The equation used was sigmoidal dose–response (variable slope), *Y* = bottom + (top − bottom)/(1 + 10 ((logIC_50_ − *X*) × Hill Slope)). Reported IC_50_ values were calculated based on the averages of three independent dataset. Assay robustness and data quality was assessed by *Z*′ > 0.5 (*Z*′ = 1 − (3 × (SD high + SD low)/(mean high − mean low))) and activity reproducibility of reference compound. WEHI‐600 (PMV) or WM382 (PMIX/PMX) were included as reference compounds in every experiment to monitor the assay performance. Any drift in activity greater than threefold from the average acceptable range and the experiment was rejected and repeated.

### *P. falciparum* growth inhibition assays

The ability of compounds to block growth of blood stage *P. falciparum* parasites was determined using a growth inhibition assay (GIA) as described [14]. Compound potency was assessed with 10 point, threefold dilution series starting at 10 μm in duplicate per experiment and repeated in three independent experiments. Data were normalized to percent viability relative to 0.1% DMSO (high growth control) and 2.5 μm chloroquine (low growth control). EC50 values (relative inflection of dose–response curve) were calculated using a nonlinear regression four‐parameter fit analysis in dotmatics 5.3 and spotfire 7.11.1 software. The equation used was sigmoidal dose–response (variable slope), *Y* = bottom + (top − bottom)/(1 + 10 ((logEC50 − *X*) × Hill Slope)). Reported EC50 values were calculated on the average of three independent dataset. Assay robustness and data quality was assessed by *Z*′ > 0.5 (*Z*′ = 1 − (3 × (SD high + SD low)/(mean high − mean low))) and activity reproducibility of reference compound WM382, which was included in every experiment. Any drift in WM382 activity greater than threefold from the average acceptable range and the experiment was rejected and repeated.

### Protein expression and purification

*Plasmodium vivax* PMX (PvPMX) and *P. vivax* PMV (PvPMV) were produced as previously described [18, 29]. Briefly, PvPMX (residues H27‐E545) was codon‐optimized for insect cell expression (Bioneer), then cloned into an insect cell expression vector bearing an N‐terminal gp67 signal peptide and C‐terminal fusion tag comprised of a tobacco etch virus (TEV) protease‐cleavage site and a FLAG tag. This was expressed in Sf21 insect cells. An N‐terminally processed form of the expected recombinant zymogen was purified from cell supernatant using anti‐FLAG M2‐agarose (Sigma‐Aldrich St Louis, MO, USA). Pooled fractions were concentrated and further purified using Gel‐filtration chromatography (Superdex 75; GE Life Sciences) in 20 mm Tris, pH 7.2, 100 mm NaCl, which resulted in a pure and stable protein that was concentrated for crystallization and other biophysical and biochemical characterizations. PvPMV (residues R35–R476) was prepared similarly, but the anti‐FLAG M2 recombinant protein concentrate was further purified using Gel‐filtration chromatography (Superdex 75; Cytiva Marlborough, MA, USA) in 20 mm HEPES, pH 7.2, 100 mm NaCl, and 0.2 mm DTT. This produced pure and stable protein that was concentrated for crystallization.

### Crystallization trials and protein structure analysis

PvPMV was cocrystallized with WM36 in 20% (w/v) PEG3350, 0.25 m Na_2_HPO_4_/KH_2_PO_4_, pH 6.88 and incubated at 20 °C. Crystals were frozen in well solution supplemented with 20% (v/v) ethylene glycol. PvPMV was cocrystallized with WM48 in 0.1 m SPG, pH 9 (succinic acid/sodium dihydrogen phosphate/glycine), 25% (w/v) PEG 1500, 12% (w/v) inositol, 0.2 mm DTT and 1 mm Anderson–Evans polyoxotungstate and crystals were frozen in a well solution supplemented with 15% (v/v) glycerol. As previously described, PvPMX apo was crystallized in 0.1 m SPG (pH 6 (succinic acid/sodium dihydrogen phosphate/glycine), 25% (w/v) PEG 1500, 30% (v/v) MPD (+/−)‐2‐methyl‐2,4‐pentanediol) and 1 mm TEW (Anderson–Evans polyoxotungstate) [18]. Crystals were frozen in a solution supplemented with the following cryoprotectants: PvPMV–WM36‐20% (v/v) ethylene glycol, PvPMV–WM48 and PvPMXapo = 15% (v/v) glycerol.

Data were collected at the Australian Synchrotron beamline MX2 at 100 K and processed with xds [41, 42], pointless [41] and aimless [43]. The PvPMX apo structure was determined by molecular replacement with the Autorickshaw server [44] using PvPMX–WM382 structure (7TBD.pdb) as the search model. PvPMV–WM36 and PvPMV–WM48 were determined by molecular replacement with the Autorickshaw server [44] using PvPMV‐WEHI‐842 structure (4ZL4.pdb). Further rounds of building and refinement with coot [45] and phenix [46] yielded the final model. The OMIT diagram was generated using Phenix composite Omit Map. Geometrical restraints for the ligands were generated using the grade webserver [47]. pymol Molecular Graphics System software version 2.5.0 was used to visualize protein structures [48]. Structures have been deposited in the Protein Data Bank (https://www.rcsb.org/) with PDB ID 8TYF, 8TYG, and 8TYH. Data collection and refinement statistics can be viewed in Table S1.

### Protein modeling

clc drug discovery workbench software (version 2.5.1) was used to model analogs WM396 and WM447 to the X‐ray structure of PfPMX (PDB: 7TBC) as described previously [18]. The structure of PvPMV–WM48 was aligned and overlaid with PfPMX providing a template for modeling and positioning the IPF scaffold. The IP head group on the IPF analogs was fixed during the modeling process. This method detects various flexible ligand conformations while holding the protein as a rigid structure during modeling. The chemical structures of analogs WM396 and WM447 were independently built and then minimalized in the Workbench environment. Two possible binding modes were observed with the pyrazole analog WM447 using the rotation function and followed by minimization. The modeling was output as a pdb file and visualized in pymol Molecular Graphics System software version 2.5.0 [48].

### Chemistry—compound preparation

The procedure for the synthesis of *R/S*‐WM48 (**14**) and *R/S*‐WM447 (**28**) (illustrated in Figs S7 and S8) is detailed in the Supplementary Information section. Other compounds used in this research were generated using the same synthetic protocols. Analytical data for these compounds are outlined in Table S2.

## Conflict of interest

ANH, AFC, JAM, and DBO have patents related to this manuscript but that do not specifically include compounds in this manuscript: Antimalarial Agents PCT/CN2019/100781.

## Author contributions

ANH designed and performed *in vitro* studies, solved structures, and drafted the manuscript. BES designed WM‐compounds and drafted manuscript. GA and SR designed compounds. AN and KJ performed and interpreted biochemical assays. SS solved structures. PC solved structures. HW and JAM designed and synthesized compounds. DBO managed project and interpreted data. AFC managed the project, designed experiments and drafted the manuscript. All authors read and edited the manuscript.

## Peer review

The peer review history for this article is available at https://www.webofscience.com/api/gateway/wos/peer‐review/10.1111/febs.70038.

## Supporting information


**Fig. S1.** Structures for the S2 loop of the PvPMV–WM48 complex.
**Fig. S2.** Comparison of surfaces at the roof of the S_1_ pocket in the structures for PvPMVWM36 (PDB: 8TYF) and PvPMVWM48 (PDB: 8TYG).
**Fig. S3.** Comparison of the PfPMXapo/PvPMXapo (PDB: 7TBB/8TYH) and the PvPMXapo/PvPMVWM48 (PDB: 8TYH/8TYG) S_1_ roof pocket surfaces.
**Fig. S4.** Prediction of the positioning of compound WM396 in the S_1_ roof pocket for PfPMX and PvPMV.
**Fig. S5.** Prediction of the positioning of compound WM447 in the S_1_ roof pocket for PfPMX and PvPMV.
**Fig. S6.** Identification of a cluster of hydrophilic residues conserved within the S_2_′ pockets of PMX and PMV and proximal to the binding position of the IPF scaffold.
**Fig. S7.** General synthetic pathway A of 6‐(3‐(4‐chlorophenyl)pyridin‐2‐yl)‐7a‐(2,5‐difluorophenyl)‐2‐imino‐3‐methylhexahydro‐1H‐pyrrolo[3,4‐d]pyrimidin‐4(4aH)‐one (**14**).
**Fig. S8.** General synthetic pathway B of 7a‐(2,5‐difluorophenyl)‐2‐imino‐3‐methyl‐6‐(4‐phenyl‐1*H*‐pyrazol‐3‐yl)hexahydro‐1*H*‐pyrrolo[3,4‐*d*]pyrimidin‐4(4a*H*)‐one (*R/S*‐WM447, **28**).
**Table S1.** Data collection and refinement statistics.
**Table S2.** LCMS and ^1^H‐NMR for representative final compounds.

## Data Availability

The data that support the findings of this study are available within the article and its Supporting Information.
