# Supplementary material for: Structure–activity analysis of imino‐pyrimidinone‐fused pyrrolidines aids the development of dual plasmepsin V and plasmepsin X inhibitors
Source: FEBS J. 2025 Mar 4;292(11):2843–64. doi: 10.1111/febs.70038 (PMC12138161; doi:10.1111/febs.70038)
Supplement: Supplementary file 1 — Fig. S1. Structures for the S2 loop of the PvPMV–WM48 complex. Fig. S2. Comparison of surfaces at the roof of the S1 pocket in the structures for PvPMVWM36 (PDB: 8TYF) and PvPMVWM48 (PDB: 8TYG). Fig. S3. Comparison of the PfPMXapo/PvPMXapo (PDB: 7TBB/8TYH) and the PvPMXapo/PvPMVWM48 (PDB: 8TYH/8TYG) S1 roof pocket surfaces. Fig. S4. Prediction of the positioning of compound WM396 in the S1 roof pocket for PfPMX and PvPMV. Fig. S5. Prediction of the positioning of compound WM447 in the S1 roof pocket for PfPMX and PvPMV. Fig. S6. Identification of a cluster of hydrophilic residues conserved within the S2′ pockets of PMX and PMV and proximal to the binding position of the IPF scaffold. Fig. S7. General synthetic pathway A of 6‐(3‐(4‐chlorophenyl)pyridin‐2‐yl)‐7a‐(2,5‐difluorophenyl)‐2‐imino‐3‐methylhexahydro‐1H‐pyrrolo[3,4‐d]pyrimidin‐4(4aH)‐one (14). Fig. S8. General synthetic pathway B of 7a‐(2,5‐difluorophenyl)‐2‐imino‐3‐methyl‐6‐(4‐phenyl‐1H‐pyrazol‐3‐yl)hexahydro‐1H‐pyrrolo[3,4‐d]pyrimidin‐4(4aH)‐one (R/S‐WM447, 28). Table S1. Data collection and refinement statistics. Table S2. LCMS and 1H‐NMR for representative final compounds. [file FEBS-292-2843-s001.docx]

*Supplementary Information*

1

**Structure-activity analysis of fused pyrrolidine imino pyrimidinones toward**

**the development of dual Plasmepsin V and Plasmepsin X inhibitors**

Anthony N. Hodder,^1,2^ Brad E. Sleebs,^1,2^ Greg Adams^3^, Sina Rezazadeh,^3^ Anna Ngo,^1,2^ Kate E.

Jarman,^1,2^ Stephen Scally,^1,2^ Peter Czabotar,^1,2^ Hongwu Wang,^3^ John A. McCauley,^3^ David B.

Olsen,^3^ Alan F. Cowman,^1,2,*^

^1^ The Walter and Eliza Hall Institute of Medical Research, Parkville 3052, Australia.

^2^ Department of Medical Biology, The University of Melbourne, Parkville 3010, Australia.

3

Merck & Co., Inc., 770 Sumneytown Pike, West Point, PA 19486, USA


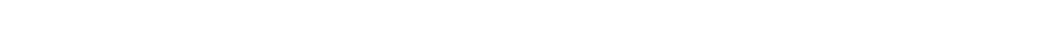


*Supplementary Information*

2

**Supplementary Figures and Tables.**

**Table S1. Data collection and refinement statistics**

f

f

f

**Pv-PMX-apo**

**PvPMV-WM36**

**PvPMV-WM48**

**Beamline**

**MX2**

**MX2**

**MX2**

**Wavelength (Å)**

**Space group**

**Cell dimensions**

***a,b,c* (Å)**

**α, β, γ (°)**

**Resolution (Å)^a^**

**0.953732**

**P 63**

**0.953700**

**I 2 2 2**

**0.953732**

**I 2 2 2**

**136.3, 136.3, 46.0**

**90, 90, 120**

**44.61-1.83(1.87-**

**1.83)**

**60.72, 81.85, 192.88**

**90, 90, 90**

**48.77-2.59 (2.69-**

**2.60**)

**63.8, 87.0, 192.5**

**90, 90, 90**

**39.64-1.64(1.70-1.64)**

**No. molecules in ASU**

**No. observations**

**No. unique observations**

**Multiplicity**

**R_merge_ (%)^b^**

**R_pim_ (%)^c^**

**1**

**1**

**1**

**444,849 (28,428)**

**43,343 (2,639)**

**10.3 (10.8)**

**9.9 (148.5)**

**3.2 (47.1)**

**16.1 (1.80)**

**100.0 (71.2)**

**100.0 (100.0)**

**121,461 (11,570)**

**15,265 (1,462)**

**8.0 (7.9)**

**13.0 (112.8)**

**4.9 (42.2)**

**12.2 (1.5)**

**99.8 (70.7)**

**99.5 (98.0)**

**444,290 (22,647)**

**65,130 (3,146)**

**6.8 (7.2)**

**5.9 (118.5)**

**2.5 (47.1)**

**14.9 (1.7)**

**99.8 (84.4)**

**99.0 (97.9)**

**<I/σ I>**

**CC_½_**

**Completeness (%)**

**Refinement Statistics**

**Reflections (work)**

**Reflections (test)**

**Non-hydrogen atoms**

**Macromolecule**

**Water**

**43,327**

**2,187**

**2,825**

**2,642**

**132**

**15,251**

**764**

**3,338**

**3,237**

**36**

**65,042**

**3,328**

**3,753**

**3,347**

**278**

**Heteroatom**

**51**

**91**

**160**

**R_work_^d^ / R_free_**

**e**

**22.82/25.16**

**18.28/23.99**

**17.85/21.08**

**Rms deviations from ideality**

**Bond lengths (Å)**

**Bond angle (°)**

**0.002**

**0.47**

**0.002**

**0.55**

**0.005**

**0.71**

**Ramachandran plot**

**Favoured regions (%)**

**Allowed regions (%)**

**B-factors (Å^2^)**

**97.57**

**2.43**

**93.18**

**6.82**

**97.75**

**2.25**

**Wilson B-value**

**31.24**

**44.03**

**43.72**

**68.00**

**40.86**

**55.52**

**64.79**

**64.65**

**78.35**

**52.74**

**29.10**

**41.77**

**40.67**

**59.03**

**47.07**

**Average B-factors**

**Average macromolecule**

**Average heteroatom**

**Average water molecule**

^a^ Values in parentheses refer to the highest resolution bin.

^b^ R_merge_ = Σ_hkl_ Σ_i_ | I_hkl, i_ - <I_hkl_ > | / Σ_hkl_ <I_hkl_

^c^ R_pim_ = Σ_hkl_ [1/(N – 1)]_1/2_ Σ_i_ | I_hkl, i_ - <I_hkl_ > | / Σ_hkl_ <I_hkl_

^d^ R_work_ = (Σ | |F | − |F | |) / (Σ | |F |) - for all data except as indicated in footnote e.

>

>

o

c

o

^e^ 5% of data were used for the R_free_ calculation

f PDB code for PvPMXapo=8TYH, PDB code for PvPMVWM36=8TYF and PDB code for PvPMVWM48=8TYG.


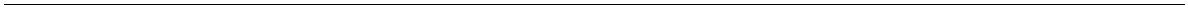

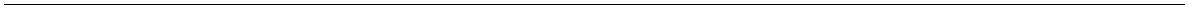

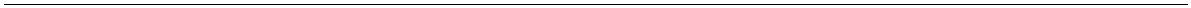


*Supplementary Information*

3

**Fig. S1.** Structures for the S2 loop of the PvPMV-WM48 complex. (A) A cartoon stick structure

of the S2 loop (**134**EYMQSYCEGSQISGF**148**) in the PvPMVWM48 complex. The cartoon

depicts the alternate positions of residues Q137-I145 (gold) due to dual occupancy of sites within

the electron density map. A maximum 5 Å deviation (red dash) in position is observed for residue

E141. The position of E141 in each conformer of the S2 loop is defined by A or B. (B) Electron

density (Omit) of the S2 loop of PvPMV, 2Fo-2Fc density contoured at 1.0 σ with mesh

representing electron density, colored gold in the area where density distribution is sufficient to

accommodate the model (residues 137Q-I145) in two alternative locations within the S2 loop

structure. (C) Residues involved in Van der Waals surface interactions (<4Å) between PvPMV

and WM48 in the dual occupied structure (WM48_dual occ) and between the individual PvPMV


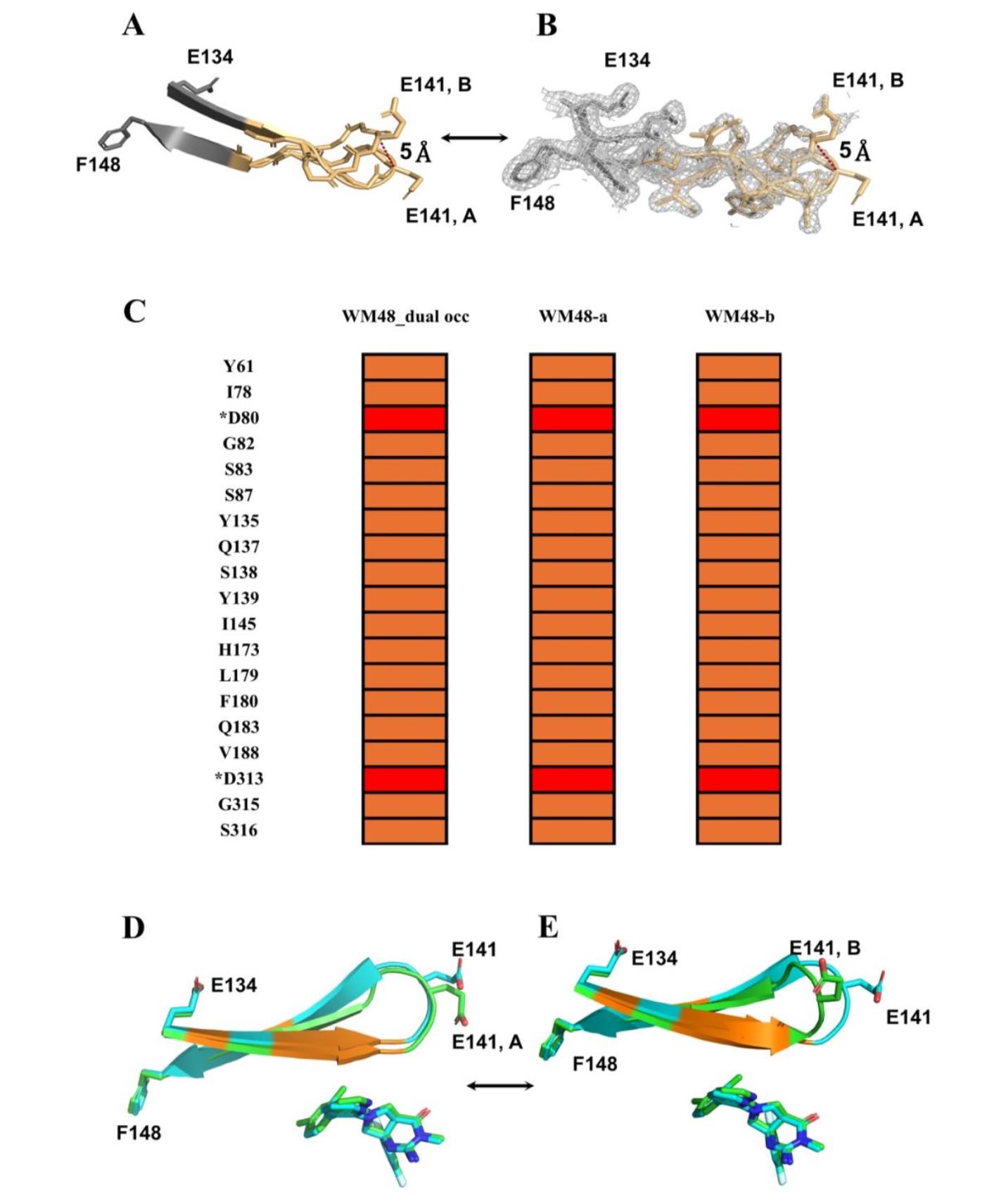


*Supplementary Information*

4

S_2_ loop conformer structures (A and B) and WM48 (shown in (A)). Interactive residues are color-

coded as orange = residues that interact with WM48, red = the location of active site aspartic acid

residues (and interactive). The interactive profiles for each were found to be the same. (D)

Schematic showing overlaid S_2_ loops from the structures of PvPMVWM36 (cyan) and

PvPMVWM48 (conformer A, green). Models for WM36 and WM48 were obtained from the

aligned structures for PvPMVWM36 (PDB: 8TYF) and PvPMVWM48 (PDB: 8TYG) and placed

to assist with orientation. The orange coloration indicates the identical locations of <4Å Van der

Waals interactions between PvPMV and both WM36 and WM48. (E) as per (D) except a

schematic showing overlaid S_2_ loops from the structures of PvPMV-WM36 (cyan) and PvPMV-

WM48 (conformer B, green). Although maintaining the same interactions with WM48 (orange),

the base of the S_2_ loop of conformer B is turned further away from the catalytic cleft and is less

like the structure for the S_2_ loop for PvPMV complexed to WM36 than conformer A. For these

reasons conformer A was used as the representative structure for PvPMV in complex with WM48

for images. For all panels the PvPMV-WM48 structure was determined by molecular replacement

with the Autorickshaw server [1] using PvPMV-WEHI-842 structure (4ZL4.pdb). Additional

rounds of building and reﬁnement with Coot [2] and Phenix [3] yielded the ﬁnal model.

*Supplementary Information*

5

**Fig. S2.** Comparison of surfaces at the roof of the S_1_ pocket in the structures for PvPMVWM36

(PDB:8TYF) and PvPMVWM48 (PDB:8TYG). (A). Side view into the catalytic cleft of PvPMV

(from the S’ side of the cleft. Aligned carbon main chains and inhibitors are shown as cartoon

structures (PvPMVWM36=green and PvPMVWM48=cyan, the 2-chloro and 4-chloro moieties

of WM36 and WM48, respectively, are both colored in wheat). Surfaces of the relevant residues

participating in the formation of the roof for the S_1_ pocket that interacts with each inhibitor are

represented by a mesh (PvPMVWM36=green and PvPMVWM48=grey). Residues involved in

the formation of the shown mesh are S87, Q137, I145 and V188. The position of the S_2_ loops for

each structure, which are found at the front of the catalytic cleft, are indicated for orientation. (B).

As per (A) but with a 180˚ anticlockwise rotation about the vertical axis, enabling the view to be

taken from the S side of the cleft. In the top RHS corner the side chains of H173 and S87, which

interact with the 4-chloro atom of WM48, can be seen. (C). Magnified version of (A) with each

main chain cartoon removed for clarity. The position of the 2-chloro and 4-chloro substitution in

WM36 and WM48 respectively is indicated by an arrow. (D) Magnified version of (B) with each

main chain cartoon removed for clarity. The position of the 2-chloro and 4-chloro substitution in

WM36 and WM48 respectively is indicated by an arrow. The surfaces for the roof of the S_1_ pocket

in each structure are observed to be very similar even with the positional change of the chlorine

atom. For all panels the PvPMV-WM36 and *Pv*PMV-WM48 structures were determined by

molecular replacement with the Autorickshaw server [1] using PvPMV-WEHI-842 structure

(4ZL4.pdb). Further rounds of building and reﬁnement with Coot [2] and Phenix [3] yielded the

ﬁnal model.


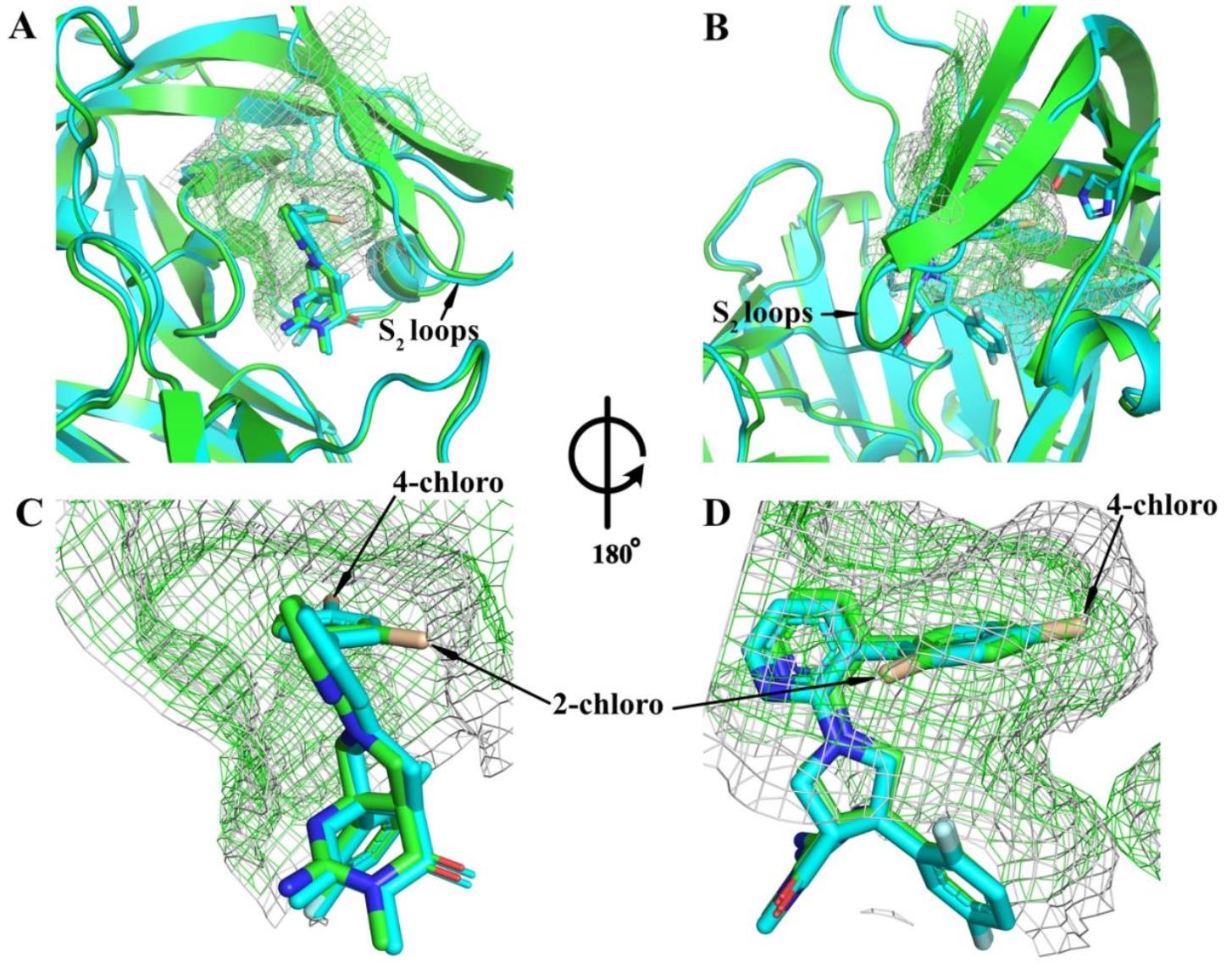


*Supplementary Information*

6

**Fig. S3**. Comparison of the PfPMXapo/PvPMXapo (PDB:7TBB/8TYH) and the

PvPMXapo/PvPMVWM48 (PDB:8TYH/8TYG) S_1_ roof pocket surfaces. (A). Side view into the

aligned catalytic clefts of PfPMXapo and PvPMXapo (from the S’ side of the cleft, side view 1).

Aligned carbon main chains are shown as cartoon structures (PfPMXapo=magenta and

PvPMXapo=light grey). Surfaces of the relevant residues participating in the formation of the

roof for the S_1_ pocket are represented by mesh (PfPMXapo=magenta and PvPMXapo=light grey).


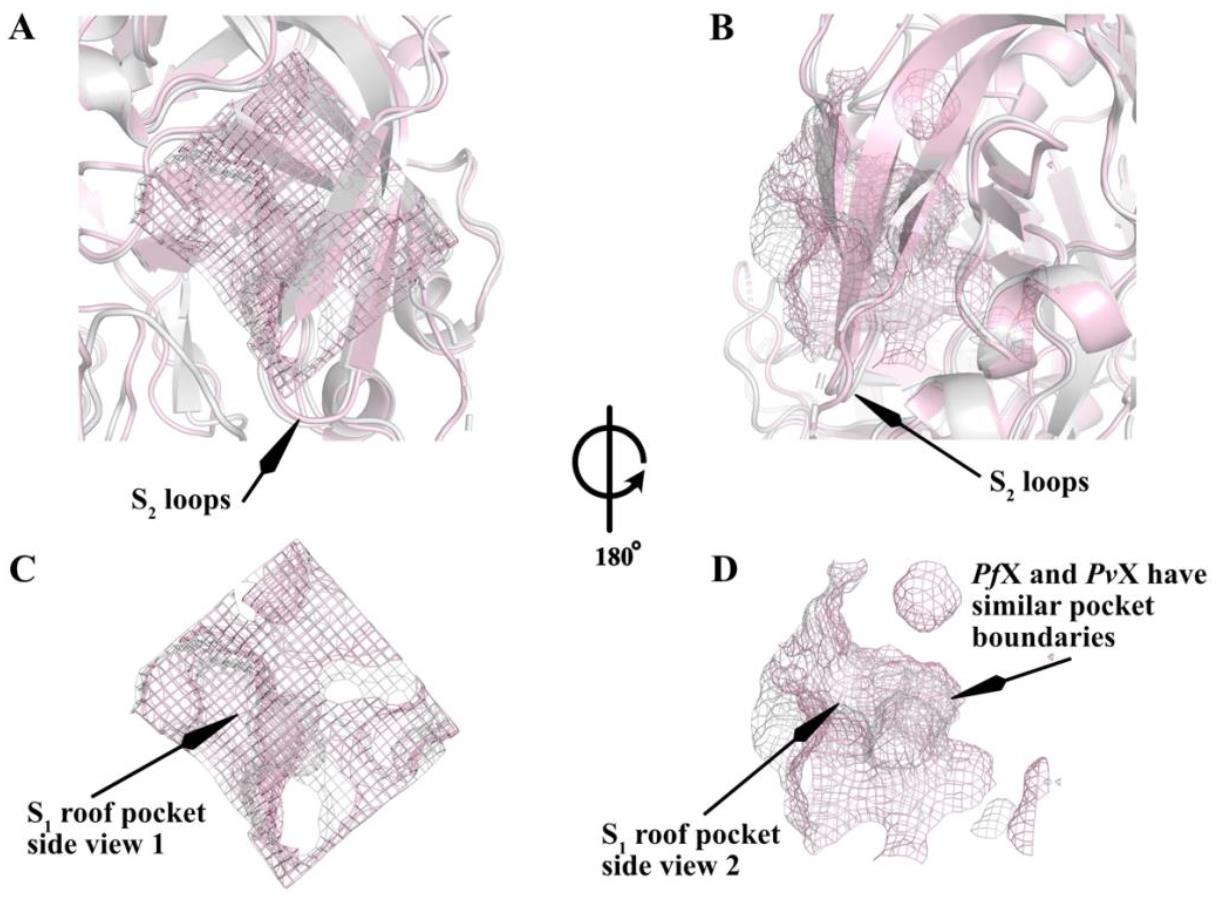

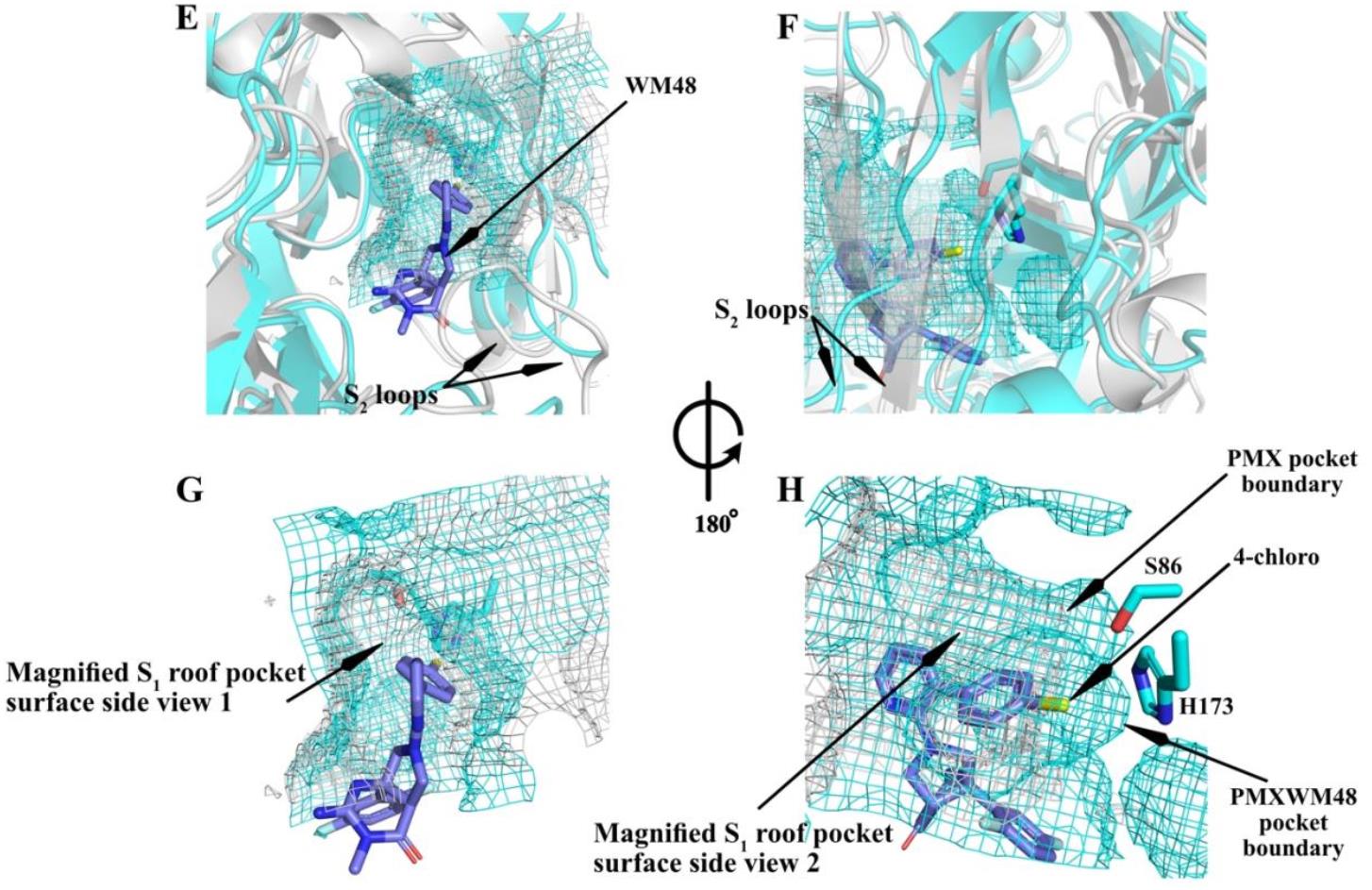


*Supplementary Information*

7

Residues involved in the formation of the shown mesh are PfPMXapo= W273, I309, I316, I363

and PvPMXapo=W238, I274, I281, I327. The position of the S_2_ loops for each structure, which

are found at the front of the catalytic cleft, are indicated for orientation. (B). As per (A) but with

a 180˚ anticlockwise rotation about the vertical axis, enabling the view to be taken from the S

side of the cleft (side view 2). (C). Magnified version of (A) with each main chain cartoon

removed for clarity. (D). Magnified version of (B) with each main chain cartoon removed for

clarity. (E). Side view into the aligned catalytic clefts of PvPMXapo and PvPMVWM48 (from

the S’ side of the cleft, side view 1). Aligned carbon main chains and compound WM48 are shown

as cartoon structures (PvPMXapo=light grey and PvPMXWM48=cyan and WM48 shown with

light purple carbon atoms, red oxygen atoms, blue nitrogen atoms and a yellow chlorine atom).

Surfaces of the relevant residues participating in the formation of the roof for the S_1_ pocket are

represented by mesh (PvPMXapo=light grey and PvPMVWM48=cyan). Residues involved in the

formation of the shown mesh are PvPMXapo = W273, I274, I281, I327 and PvPMXapo=S87,

Q137, I145, V188. The position of the S_2_ loops for each structure, which are found at the front of

the catalytic cleft, are indicated for orientation. (F). As per (E) but with a 180˚ anticlockwise

rotation about the vertical axis, enabling the view to be taken from the S side of the cleft (side

view 2). (G). Magnified version of (E) with each main chain cartoon removed for clarity. (H).

Magnified version of (F) with each main chain cartoon removed for clarity. In this diagram, the

difference in the depth of the S_1_ pockets is visible and indicates that WM48 is too large to be

optimally accommodated in the PMX structure. PvPMV S86 and H173 interact with the 4-chloro

moiety in WM48 and are shown for orientation. For all panels the PvPMX apo structure was

determined by molecular replacement with the Autorickshaw server [1] using PvPMX-WM382

structure (7TBD.pdb) as the search model. *Pv*PMV-WM48 was determined by molecular

replacement with the Autorickshaw server [1] using PvPMV-WEHI-842 structure, 4ZL4.pdb).

Further rounds of building and reﬁnement with Coot [2] and Phenix [3] yielded the ﬁnal model.

*Supplementary Information*

8

**Fig. S4**. Prediction of the positioning of compound WM396 in the S_1_ roof pocket for PfPMX and

PvPMV. (A). Side view into the catalytic cleft of PfPMX (from the S’ side of the cleft, side

view 1). Main chain carbons are shown as cartoon structures (PfPMX=salmon). Carbon atoms of

compound WM396 are displayed in grey, while nitrogen atoms are colored blue, fluorine atoms

are colored light blue, oxygen atoms are colored red, sulfur atoms are colored yellow the 3-chloro

substitution of the thiophene ring is colored green and the thiophene ring carbon atoms are colored

magenta to assist with identification of this moiety. The residues participating in the surface for

the roof for the S_1_ pocket are represented by mesh (PfPMX=salmon). Residues involved in the


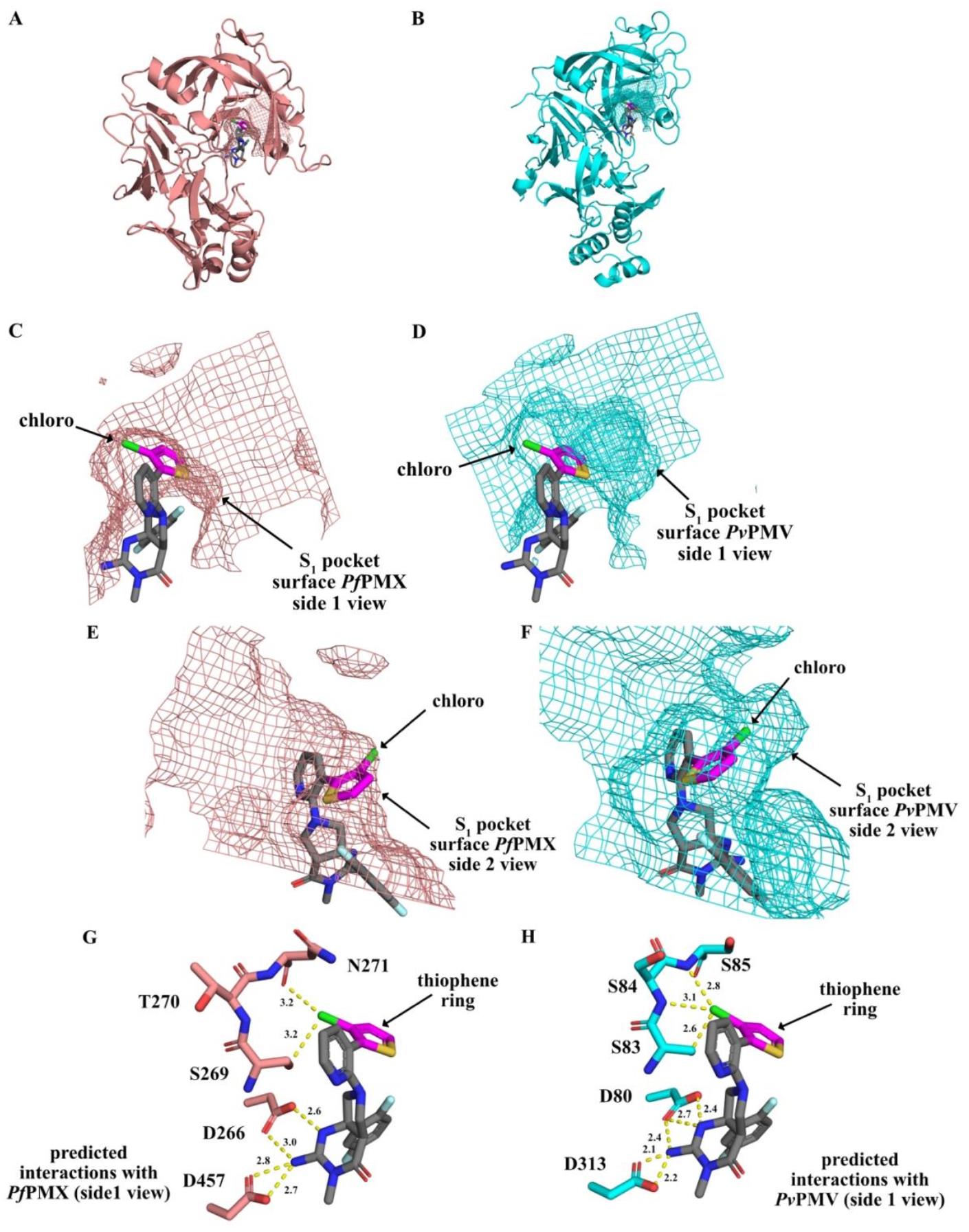


*Supplementary Information*

9

formation of the mesh surface are PfPMX= W273, I309, I316, I363. CLC Drug Discovery

Workbench software (version 2.5.1) was used to model analog WM396 to the X-ray structure of

PfPMX (PDB:7TBC). The modeled structure is termed PfPMX-model for identification. (B). As

per (A) but the positioning of compound WM396 in PvPMV (PDB:8TYG) was obtained via

alignment to the modeled structure for PfPMX see (A). Main chain carbons are shown as cartoon

structures (PvPMV=cyan). The mesh surface (cyan) for the roof of the S_1_ pocket was determined

from PvPMV= S85, Q137, I145 and V188. (C). Magnified version of (A) showing compound

WM396 and the surface (mesh) for the roof of the S_1_ pocket. The main chain carbon cartoon was

removed for clarity. (D). Magnified version of (B) showing compound WM396 and the surface

(mesh) for the roof of the S_1_ pocket. The main chain carbon cartoon was removed for clarity. (E).

A side view into the catalytic cleft of PfPMX from the S side of the cleft obtained by a

180˚clockwise rotation about the vertical axis in (C) (side view 2). The thiophene ring of

compound WM396 is predicted to fit more snuggly into the S_1_ roof cavity of PfPMX than in

PvPMV in (F). (F). A side view into the catalytic cleft of PvPMX from the S side of the cleft

obtained by a 180˚clockwise rotation about the vertical axis in (D) (side view 2). (G). A

deconstructed cartoon image generated from the PfPMX-model structure showing residues that

are predicted to be near the 3-chloro substituted thiophene ring that have the potential to

participate in the formation of halogen and hydrogen bonds (yellow dashes, numbers represent

approximate bond lengths (Å)). D266 and D457 are the active site aspartic acids in PfPMX. (H).

A deconstructed cartoon image generated from the alignment of the PfPMX-model and

PvPMVWM48 structures showing PvPMV residues that are predicted to be near the 3-chloro

substituted thiophene ring that have the potential to participate in the formation of halogen and

hydrogen bonds (yellow dashes, numbers represent approximate bond lengths (Å)). D80 and

D313 are the active site aspartic acids in PvPMV. For all panels the CLC Drug Discovery

Workbench software (version 2.5.1) was used to model analogs WM396 to the X-ray structure of

PfPMX (PDB:7TBC) as described previously [4]. The structure of *Pv*PMV-WM48 was aligned

and overlaid with PfPMX providing a template for modeling and positioning the IPF scaffold.

*Supplementary Information*

10

**Fig. S5**. Prediction of the positioning of compound WM447 in the S_1_ roof pocket for PfPMX and

PvPMV. (A). Side view into the catalytic cleft of PfPMX (from the S’ side of the cleft, side view

1). Main chain carbons are shown as cartoon structures (PfPMX=salmon). Carbon atoms of

compound WM447 are displayed in grey, while nitrogen atoms are colored blue, fluorine atoms

are colored light blue, oxygen atoms are colored red, and the pyrazol ring carbon atoms are


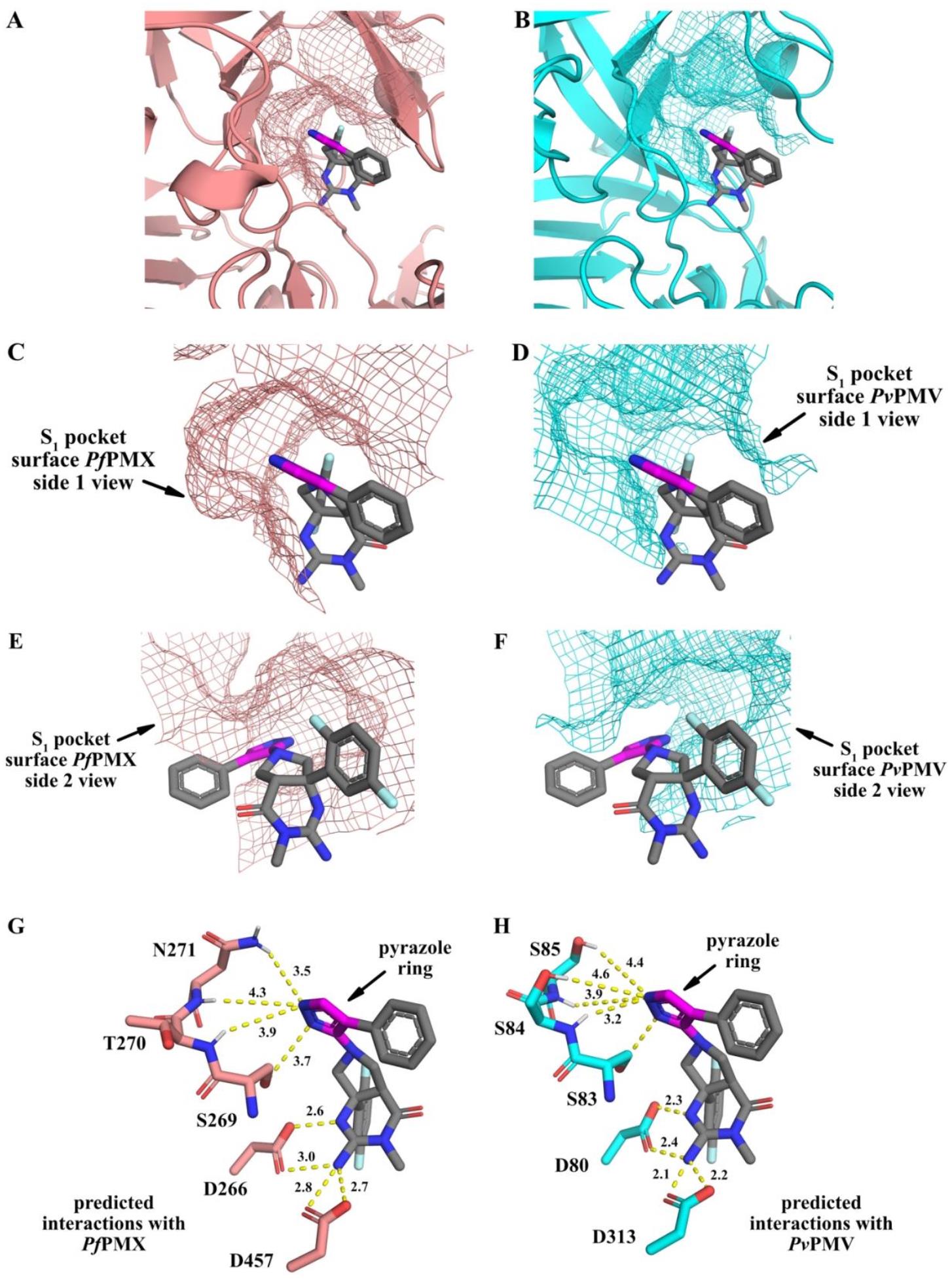


*Supplementary Information*

11

highlighted in cyan to assist with identification of this moiety. The residues participating in the

surface for the roof for the S_1_ pocket are represented by mesh (PfPMX=salmon). Residues

involved in the formation of the mesh surface are PfPMX= W273, I309, I316, I363. CLC Drug

Discovery Workbench software (version 2.5.1) was used to model analog WM447 to the Xray

structure of PfPMX (PDB:7TBC). The modeled structure is termed PfPMX-model for

identification. (B). As per (A) but the positioning of compound WM447 in PvPMV (PDB:8TYG)

was obtained via alignment to the modeled structure for PfPMX, see (A). Main chain carbons are

shown as cartoon structures (PvPMV=cyan). The mesh surface (cyan) for the roof of the S_1_ pocket

was determined from PvPMV= S85, Q137, I145 and V188. (C). Magnified version of (A)

showing compound WM447 and the surface (mesh) for the roof of the S1 pocket. The main chain

carbon cartoon was removed for clarity. (D). Magnified version of (B) showing compound

WM447 and the surface (mesh) for the roof of the S_1_pocket. The main chain carbon cartoon was

removed for clarity. (E). A side view into the catalytic cleft of PfPMX from the S side of the cleft

obtained by a 180˚clockwise rotation about the vertical axis in (C) (side view 2). (F). A side view

into the catalytic cleft of PvPMX from the S side of the cleft obtained by a 180˚clockwise rotation

about the vertical axis in (D) (side view 2). Modelling predicts the pyrazole ring of compound

WM447 interacts with a different region within the S_1_roof cavity in PfPMX and PvPMV,

compared to the thiophene ring of compound WM396 (Fig. S4), and the fit of compound WM447

to this surface is much poorer in comparison. (G). A deconstructed cartoon image generated from

the PfPMX-model structure showing residues that are predicted to be near the reactive N atoms

of the pyrazole ring and have the potential to participate in the formation of hydrogen bonds

(yellow dashes, numbers represent approximate bond lengths (Å)). D266 and D457 are the active

site aspartic acids in PfPMX. (H). A deconstructed cartoon image generated from the alignment

of the PfPMXmodel and PvPMVWM48 structures showing PvPMV residues that are predicted

to be in close proximity to the reactive N atoms of the pyrazole ring and have the potential to

participate in the formation of hydrogen bonds (yellow dashes, numbers represent approximate

bond lengths (Å)). D80 and D313 are the active site aspartic acids in PvPMV. For all panels the

CLC Drug Discovery Workbench software (version 2.5.1) was used to model analog WM447 to

the X-ray structure of PfPMX (PDB:7TBC) [4]. The structure of *Pv*PMV-WM48 was aligned and

overlaid with PfPMX providing a template for modeling and positioning the IPF scaffold. The

chemical structure of analog WM447 was independently built and then minimalized in the

Workbench environment.

*Supplementary Information*

12

**Fig. S6**. Identification of a cluster of hydrophilic residues conserved within the S_2_’ pockets of

PMX and PMV and proximal to the binding position of the IPF scaffold. (A). A view of the

catalytic cleft surfaces for the overlaid structures for *Pf*PMX-model (based on PDB:7TBC,

salmon) and *Pv*PMV (PDB:8TYG, cyan). The circle shows the location of cluster of hydrophilic

residues located in the aligned structures. The second rotomeric form of compound WM447 (see

WM447 binding mode 2 in Fig. 8) is shown in its predicted binding position within the catalytic

cleft (carbon atoms=grey, nitrogen atoms =blue and oxygen atoms=red. The pyrazole ring is

highlighted in magenta and the adjoining terminal aryl ring is shown in lime). The red arrow

shows the direction new moieties should be designed for the engagement of the hydrophilic

cluster within the S_2_’ pocket. (B) A deconstructed view showing residue side chains and main

chain amide and carbonyl groups of the hydrophilic pocket for *Pf*PMX and their orientation with

WM447 as shown in (A). Prominent residues within the hydrophilic cluster are labelled and

indicated by arrows. Please note that this panel is identical to panel E to enable side by side

comparison directly with panel (C). (C) A deconstructed view showing residue side chains and

main chain amide and carbonyl groups of the corresponding hydrophilic pocket for *Pv*PMV and

their orientation with WM447 as shown in (A). Panels (A), (B) and (C) were obtained from

overlaid structures of PDB:7TBC and 8TYG. (D). A view of the catalytic cleft surfaces for the

overlaid structures for *Pf*PMX-model (based on PDB:7TBC, salmon) and *Pv*PMX (PDB:8TYH,

light purple). See (A) for other details. (E). A deconstructed view showing residue side chains

and main chain amide and carbonyl groups of the hydrophilic pocket for *Pf*PMX and their

orientation with WM447 as shown in (D). Prominent residues within the hydrophilic cluster are

labelled and indicated by arrows. Please note that this panel is identical to panel B to enable side

by side comparison directly with panel (F). (F). A deconstructed view showing residue side chains

and main chain amide and carbonyl groups of the corresponding hydrophilic pocket for *Pv*PMX

and their orientation with WM447 as shown in (D). Panels (D), (E) and (F) were obtained from

overlaid structures of PDB:7TBC and 8TYH. Note the level of residue identity is much higher in

the aligned structures in (E) and (F) than in (B) and (C). Residues shown in (B) for *Pf*PMX-model

(salmon) are: G268, G367, F368, M371, S430, Y431, W432, E433, I455 and F456 and (C) for


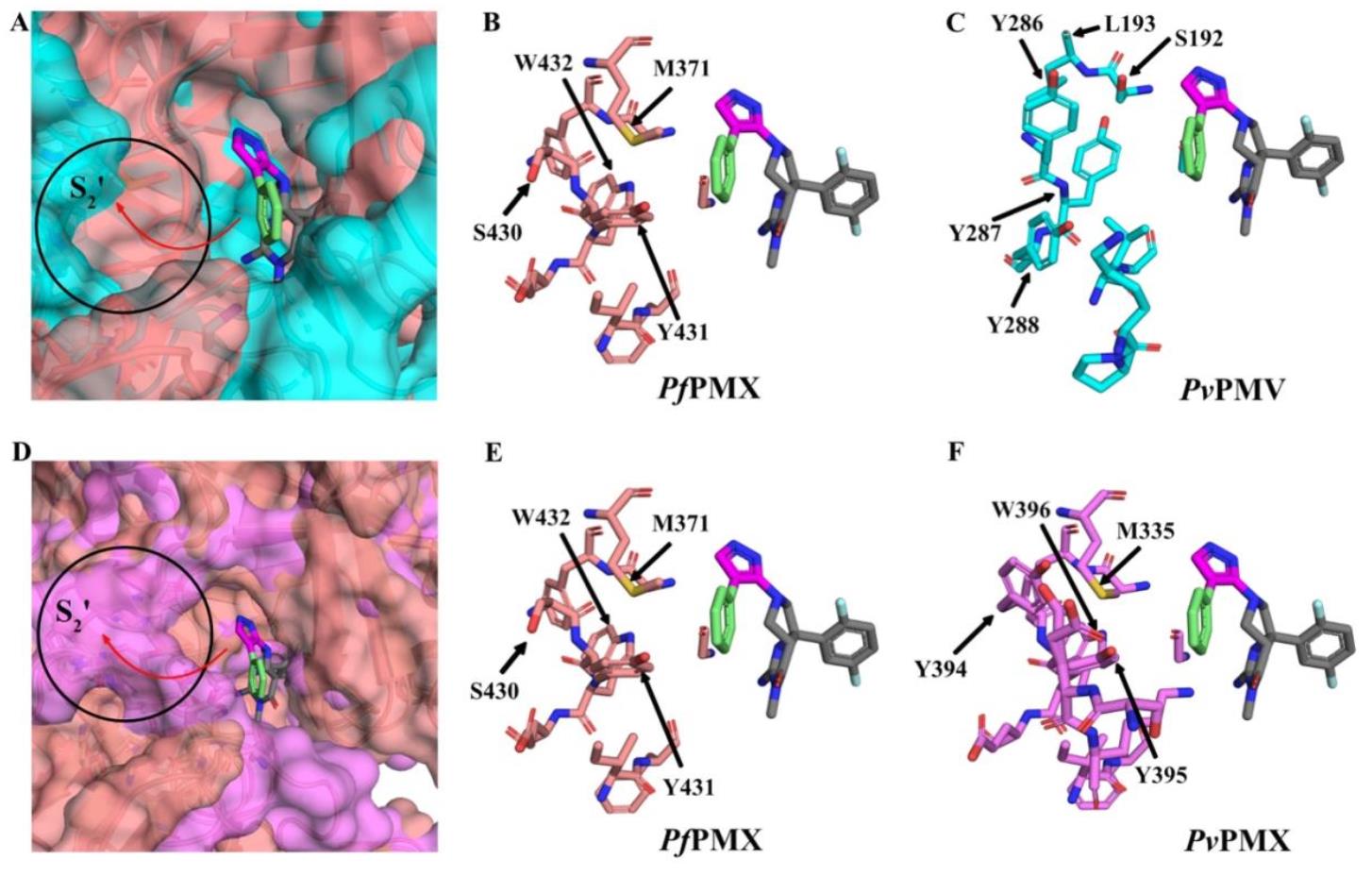


*Supplementary Information*

13

*Pv*PMV (cyan) are: G82, S192, L193, Y286, Y287, Y288, L311, V312, P438 and K437. Residues

shown in (E) for *Pf*PMX-model (salmon) are G268, G367, F368, M371, S430, Y431, W432,

E433, I455 and F456 and (F) for PvPMX (purple) are G233, F332, M335, Y394, Y395, W396,

E397, I419, F420, S496, E497 and K498. Note not all residues are labeled for simplicity. For all

panels the PvPMX apo structure was determined by molecular replacement with the

Autorickshaw server [1] using PvPMX-WM382 structure (7TBD.pdb) as the search model. The

CLC Drug Discovery Workbench software (version 2.5.1) was used to model analog WM447 to

the X-ray structure of PfPMX (PDB:7TBC) [4]. The chemical structure of analog WM447 was

independently built and then minimalized in the Workbench environment.

**Fig. S7.** General synthetic pathway A of 6-(3-(4-Chlorophenyl)pyridin-2-yl)-7a-(2,5-difluorophenyl)-

2-imino-3-methylhexahydro-1H-pyrrolo[3,4-d]pyrimidin-4(4aH)-one (**14**). The S-WM48 enantiomer was

fractionated suing liquid chromatography (**30**).


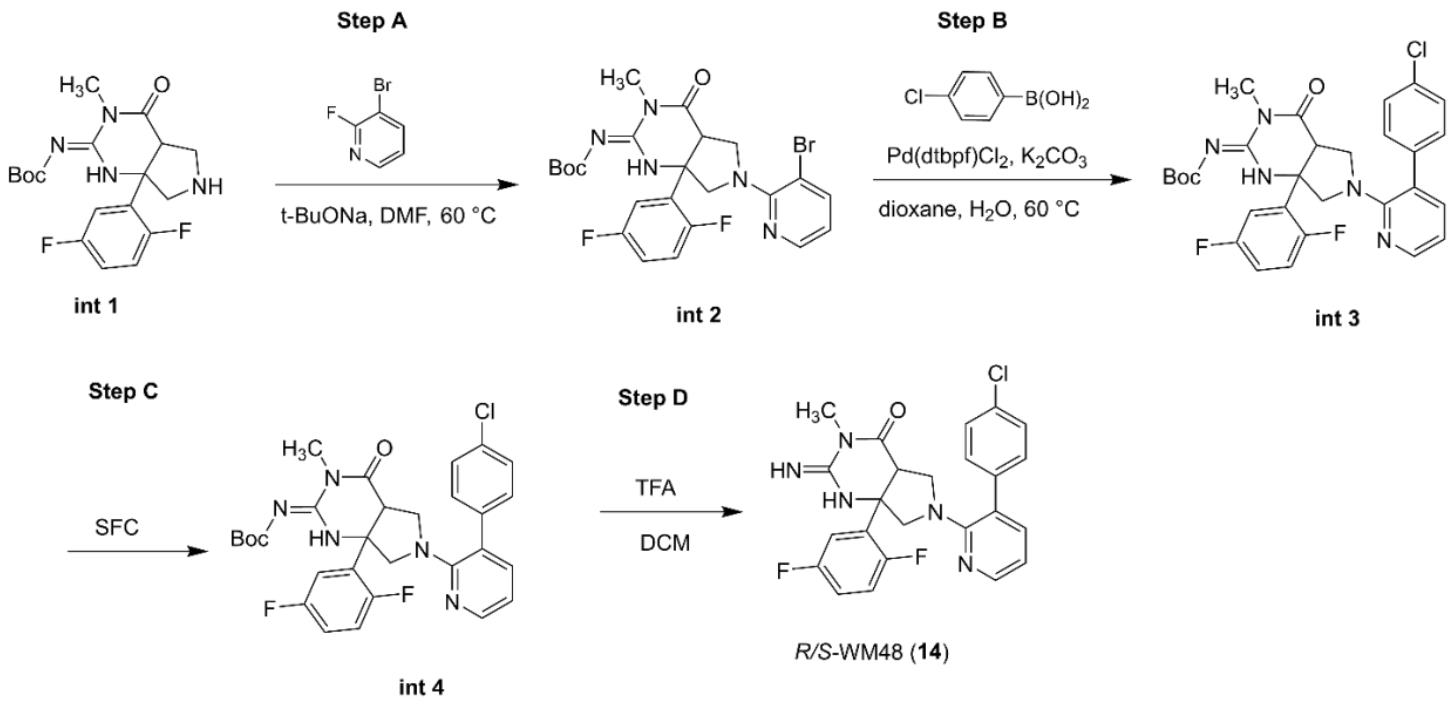


*Supplementary Information*

14

**Fig. S8.** General synthetic pathway B of 7a-(2,5-Difluorophenyl)-2-imino-3-methyl-6-(4-phenyl-1*H*-

pyrazol-3-yl)hexahydro-1*H*-pyrrolo[3,4-*d*]pyrimidin-4(4a*H*)-one (*R/S*-WM447, **28**).


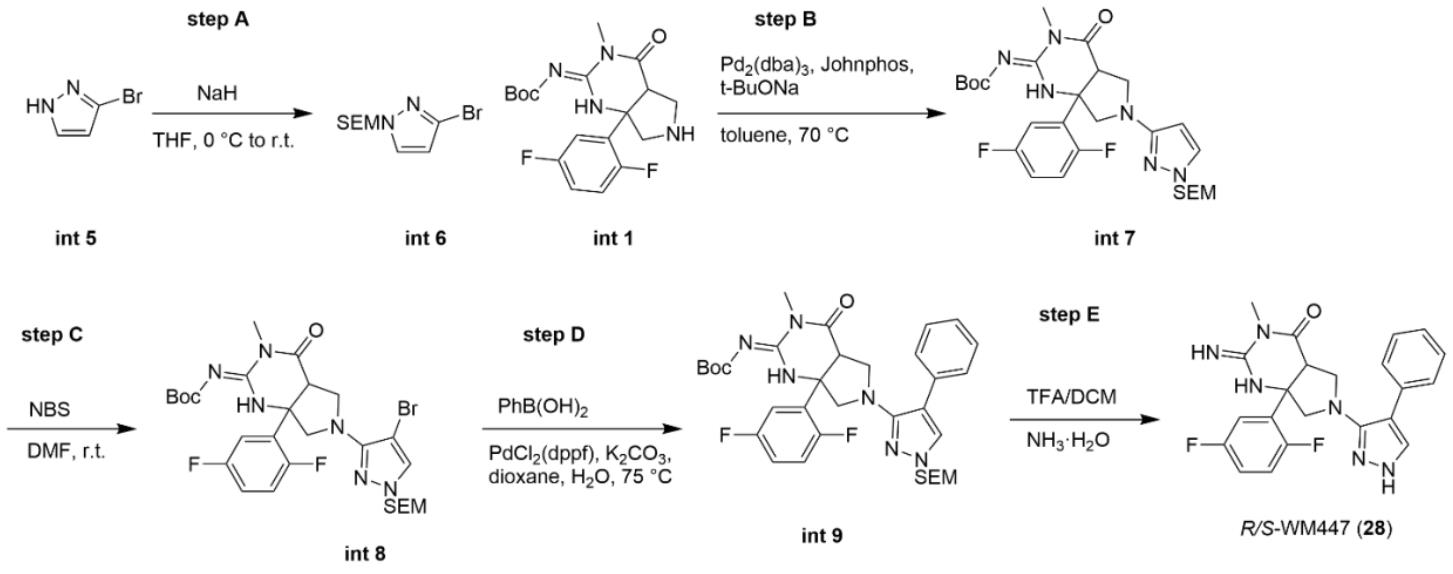


*Supplementary Information*

15

**Table S2. LCMS and ^1^H-NMR for representative final compounds.**

Compound

LCMS ^1^H-NMR

(M+1)

Compound **6**

^1^H NMR (400 MHz, methanol-d_4_) δ 8.16 (dd, *J* = 1.6, 4.8 Hz, 1H), 7.62

(dd, *J* = 2.0, 7.2 Hz, 1H), 7.32-7.45 (m, 5H), 7.19-7.27 (m, 2H), 7.02-

7.08 (m, 1H), 6.97-7.01 (m, 1H), 4.03 (t, *J* = 8.8 Hz, 1H), 3.92-3.95 (m,

1H), 3.62-3.75 (m, 3H), 3.25 (s, 3H).

434.2

468.2

468.1

*R/S-*WM36 (**8**)

*R/S*-WM48 (**14**)

^1^H NMR (500 MHz, methanol-d_4_) δ 8.19-8.27 (m, 1H), 7.36-7.60 (m,

5H), 7.21-7.31 (m, 2H), 7.10 (m, 1H), 6.97-7.02 (m, 1H), 3.91-4.18 (m,

2H), 3.55-3.79 (m, 3H), 3.29 (s, 3H).

^1^H NMR (500 MHz, methanol-d_4_) δ 8.20 (dd, *J* = 1.5, 5.0 Hz, 1H), 7.66

(dd, *J* = 1.5, 7.5 Hz, 1H), 7.43-7.51 (m, 4H), 7.22-7.32 (m, 2H), 7.09

(m, 1H), 7.02-7.04 (m, 1H), 4.08 (t, *J* = 8.5 Hz, 1H), 3.99 (dd, *J* = 2.5,

12.0 Hz, 1H), 3.67-3.82 (m, 3H), 3.29 (s, 3H).

Compound **19**

^1^H NMR (500 MHz, methanol-d_4_) δ 8.23 (dd, *J* = 2.0, 5.0 Hz, 1H), 7.65-

7.72 (m, 2H), 7.61 (d, *J* = 8.0 Hz, 1H), 7.41 (dd, *J* = 2.0, 8.0 Hz, 1H),

7.22-7.34 (m, 2H), 7.12 (m, 1H), 7.04 (dd, *J* = 5.0, 7.50 Hz, 1H), 4.09

(t, *J* = 8.5 Hz, 1H), 4.02 (dd, *J* = 2.5, 12.0 Hz, 1H), 3.84 (d, *J* = 12.0 Hz,

1H), 3.75-3.77 (dd, *J* = 8.0, 11.0 Hz, 1H), 3.64-3.70 (m, 1H), 3.30 (s,

3H).

502.1

*R/S*-WM396 (**25**)

Compound **26**

Compound **27**

Compound **29**

*R/S*-WM48 (**14**)

^1^H NMR (400 MHz, methanol-d_4_) δ 8.23 (dd, *J* = 1.6, 4.8 Hz, 1H), 7.54-

7.65 (m, 2H), 7.26 (m, 2H), 7.05-7.16 (m, 1H), 7.01 (d, *J* = 5.2 Hz, 1H),

6.92-6.93 (m, 1H), 4.02-4.14 (m, 2H), 3.81-3.84 (m, 1H), 3.66-3.74 (m,

2H), 3.28 (s, 3H).

474.1

474.1

440.2

^1^H NMR (400 MHz, methanol-d_4_) δ 8.18 (br d, *J* = 4.8 Hz, 1H), 7.72

(d, *J* = 7.6 Hz, 1H), 7.20-7.33 (m, 2H), 7.08-7.16 (m, 1H), 6.94-7.06

(m, 3H), 4.02-4.05 (m, 2H), 3.86-3.97 (m, 2H), 3.70-3.77 (m, 1H), 3.31

(s, 3H).

^1^H NMR (500 MHz, methanol-d_4_) δ 8.67 (s, 1H), 7.81 (d, *J* = 7.0 Hz,

2H), 7.45-7.46 (m, 2H), 7.33-7.39 (m, 1H), 7.30 (m, 2H), 7.12-7.20 (m,

1H), 4.17-4.19 (m, 1H), 3.83-3.88 (m, 1H), 3.79-3.80 (m, 1H), 3.72-

3.77 (m, 1H), 3.65-3.71 (m, 1H), 3.37 (s, 3H).

^1^H NMR (500 MHz, methanol-d_4_) δ 7.49 (s, 1H), 7.39 (d, *J* = 7.0 Hz,

2H), 7.32 (t, *J* = 7.5 Hz, 2H), 7.00 - 7.22 (m, 4H), 3.81-3.83 (m, 1H),

3.77 (s, 3H), 3.70-3.72 (m, 1H), 3.57 - 3.64 (m, 2H), 3.50-3.53 (m, 1H),

3.20 (s, 3H).

437.2

468.1

^1^H NMR (500 MHz, methanol -d_4_) δ 8.20 (dd, *J* = 1.5, 5.0 Hz, 1H), 7.66

(dd, *J* = 1.5, 7.5 Hz, 1H), 7.43-7.51 (m, 4H), 7.22-7.32 (m, 2H), 7.09


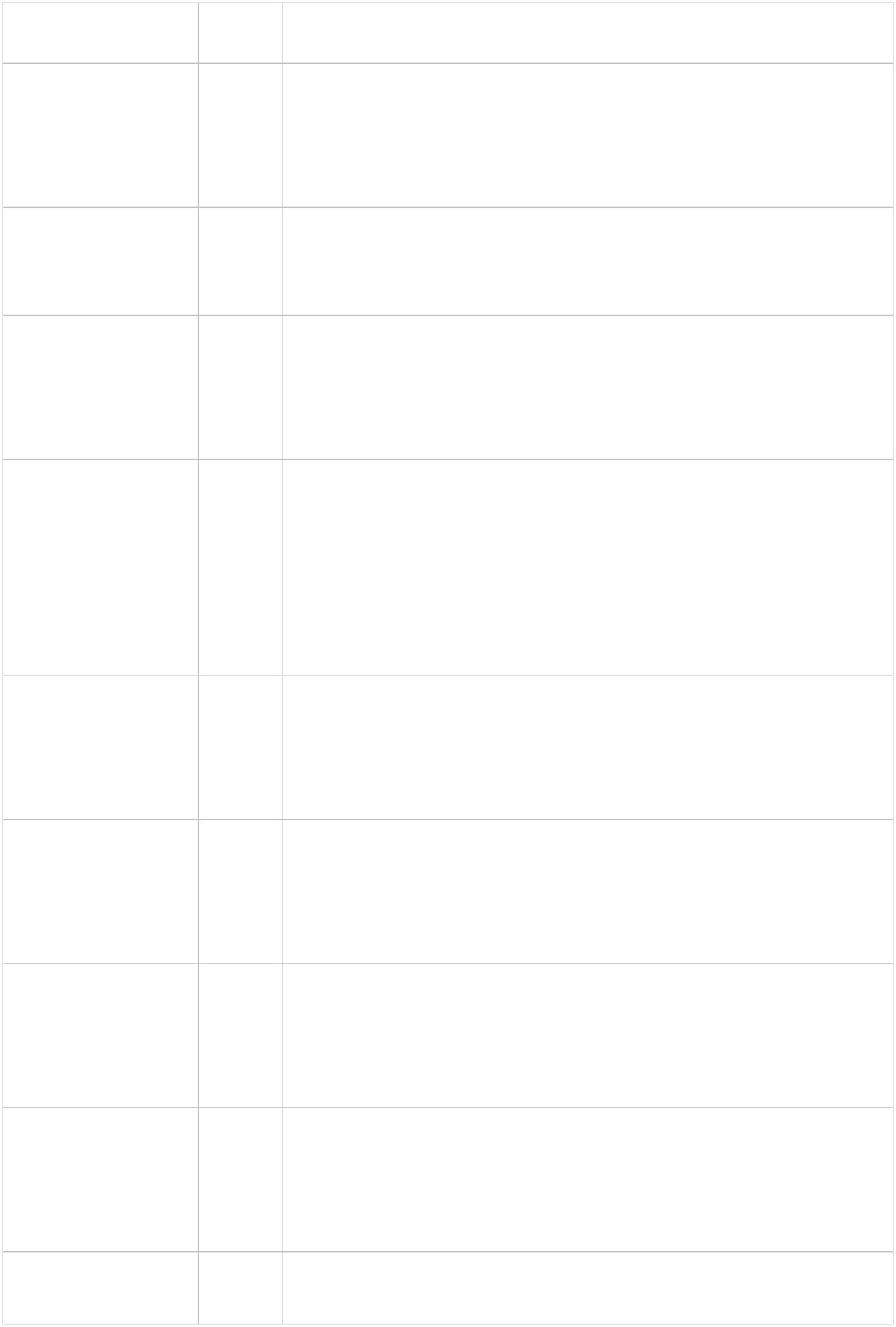


*Supplementary Information*

16

(m, 1H), 7.02-7.04 (m, 1H), 4.08 (t, *J* = 8.5 Hz, 1H), 3.99-4.02 (m, 1H),

3.67-3.82 (m, 3H), 3.29 (s, 3H).

*R*-WM48 (**31**)

^1^H NMR (500 MHz, methanol-d_4_) δ 8.19 (dd, *J* = 1.5, 5.0 Hz, 1H), 7.68

(dd, *J* = 2.0, 7.50 Hz, 1H), 7.44-7.46 (m, 4H), 7.22-7.35 (m, 2H), 7.00-

7.13 (m, 2H), 4.08 (t, *J* = 8.5 Hz, 1H), 3.98-4.00 (m, 1H), 3.75-3.83 (m,

2H), 3.68-3.75 (m, 1H), 3.29 (s, 3H).

468.1

**Chemistry experimental**

**General synthesis A:** 6-(3-(4-Chlorophenyl)pyridin-2-yl)-7a-(2,5-difluorophenyl)-2-imino-3-

methylhexahydro-1H-pyrrolo[3,4-d]pyrimidin-4(4aH)-one (**30**).

Step

A:

(E)-*tert*-Butyl

(6-(3-bromopyridin-2-yl)-7a-(2,5-difluorophenyl)-3-methyl-4-

oxohexahydro-1*H*-pyrrolo[3,4-*d*]pyrimidin-2(3*H*)-ylidene)carbamate (**int 2**).

To a mixture of 3-bromo-2-fluoropyridine (555 mg, 3.15 mmol) and sodium *tert*-butoxide (404

mg, 4.21 mmol) in DMF (5 mL) was added (E)-*tert*-butyl (7a-(2,5-difluorophenyl)-3-methyl-4-

oxohexahydro-1*H*-pyrrolo[3,4-*d*]pyrimidin-2(3*H*)-ylidene)carbamate **int 1** (400 mg, 1.052

mmol) at 25 °C under N_2_ atmosphere. Then the mixture was stirred at 60 °C for 10 h. The mixture

was quenched with water (10 mL) and extracted with EtOAc (10 mL × 3). The organic layers

were washed with brine (5 mL), dried over anhydrous Na SO , filtered, and concentrated in

2

4

vacuo. The crude was purified by preparative-TLC (SiO_2_, Pet.ether/EtOAc = 3:1) to afford **int 2**

(200 mg, 0.373 mmol, 35.5% yield) as a yellow oil. MS (ESI) *m/z:* 536.1/538.1 (M+H+). ^1^H NMR

(400 MHz, chloroform-d) δ 10.38 (br s, 1H), 8.04-8.17 (m, 1H), 7.73 (br d, *J* = 8.0 Hz, 1H), 6.90-

7.17 (m, 3H), 6.60-6.72 (m, 1H), 4.20-4.34 (m, 3H), 4.14 (br t, *J* = 10.8 Hz, 1H), 3.85-3.96 (m,

1H), 3.27 (s, 3H), 1.49 (s, 9H).

Step B: (E)-*tert*-Butyl (6-(3-(4-chlorophenyl)pyridin-2-yl)-7a-(2,5-difluorophenyl)-3-methyl-4-

oxohexahydro-1*H*-pyrrolo[3,4-*d*]pyrimidin-2(3*H*)-ylidene)carbamate (**int 3**).

A mixture of **int 2** (75 mg, 0.140 mmol), 4-chlorophenyl)boronic acid (32.8 mg, 0.210 mmol),

PdCl (DTBPF) (9.11 mg, 0.014 mmol) and K CO (58.0 mg, 0.419 mmol) in dioxane (2 mL) and

2

2

3

water (0.4 mL) was stirred at 60 °C for 2 h under N_2_ atmosphere. The mixture was quenched with

water (5 mL) and extracted with EtOAc (5 mL × 3). The organic layers were washed with brine

(5 mL), dried over Na SO , filtered, and concentrated in vacuo. The crude was purified by

2

4

preparative-TLC (SiO_2_, Pet.ether/EtOAc = 3:1) to afford **int 3** (45 mg, 0.079 mmol, 56.7% yield)

as a yellow oil. MS (ESI) *m/z:* 568.2 (M+H+).


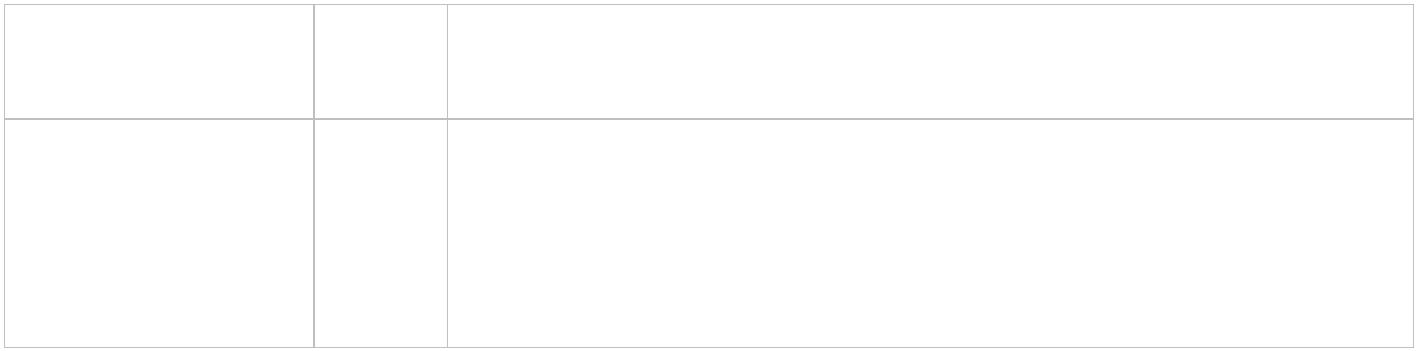

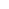


*Supplementary Information*

17

Step C: (E)-*tert*-Butyl (6-(3-(4-chlorophenyl)pyridin-2-yl)-7a-(2,5-difluorophenyl)-3-methyl-4-

oxohexahydro-1*H*-pyrrolo[3,4-*d*]pyrimidin-2(3*H*)-ylidene)carbamate (**int 4**).

Racemic **int 3** (40 mg, 0.070 mmol) was purified by chiral preparative reverse phase

chromatography (Column: Diacel Chiralpak AD-H, 250 mm × 30 mm, 5 µm; Conditions:

0.1%NH H O IPA, Begin B: 20, End B: 20; Flow rate 60 mL/min; Injections: 70) to afford the

3.

2

*R*-isomer of **int 4** (20 mg, 0.035 mmol, 50.0% yield) (Rt = 0.926 min, first eluted isomer) and *S*-

isomer of **int 4** (20 mg, 0.035 mmol, 50.0% yield) (Rt = 1.033 min, second eluted isomer). MS

(ESI) *m/z*: 568.1 (M+H+).

Step

D:

6-(3-(4-chlorophenyl)pyridin-2-yl)-7a-(2,5-difluorophenyl)-2-imino-3-

methylhexahydro-1*H*-pyrrolo[3,4-*d*]pyrimidin-4(4a*H*)-one (*S*-WM48, **30**).

A solution of the *S*-isomer of **int 4** (20 mg, 0.035 mmol) in DCM (5 mL) and TFA (1 mL) was

stirred at 15 °C for 0.2 h. LCMS showed the reaction was completed. The mixture was

concentrated and purified by preparative HPLC (Column: Agela Dura Shell C18 150 × 25 mm, 5

µm; Conditions: water (0.1%TFA)-CAN; Begin B: 30 End B: 60; Gradient time: 10 min; 100%

B hold time: 1 min; Flow rate 25 mL/min; Injections 1) to afford *S*-isomer of WM48 (**30**) as a

2,2,2-trifluoroacetate salt (15.32 mg, 0.026 mmol, 75% yield) as a white solid. MS (ESI) *m/z:*

468.1 (M+H+). ^1^H NMR (500 MHz, methanol-d_4_) δ 8.20 (dd, *J* = 2.0, 5.0 Hz, 1H), 7.63 (dd, *J* =

1.5, 7.5 Hz, 1H), 7.42-7.50 (m, 4H), 7.21-7.32 (m, 2H), 7.09 (m, 1H), 7.02 (dd, *J* = 5.0, 7.5 Hz,

1H), 4.06 (t, *J* = 8.5 Hz, 1H), 3.96-3.99 (m, 1H), 3.74-3.81 (m, 2H), 3.65-3.72 (m, 1H), 3.29 (s,

3H).

**General synthesis B:** 7a-(2,5-Difluorophenyl)-2-imino-3-methyl-6-(4-phenyl-1H-pyrazol-3-

yl)hexahydro-1H-pyrrolo[3,4-d]pyrimidin-4(4aH)-one (**28**)

Step A: 3-bromo-1-((2-(trimethylsilyl)ethoxy)methyl)-1*H*-pyrazole (**int 6**).

To a stirred solution of 3-bromo-1*H*-pyrazole **int 5** (500 mg, 3.40 mmol) in THF (10 mL) was

added NaH (163 mg, 4.08 mmol, 60% wt) at 0 °C at room temperature under N_2_ atmosphere, and

the mixture was stirred for 10 min, then SEM-Cl (0.603 mL, 3.40 mmol) was added dropwise.

The mixture was stirred at 20 °C for 4 h. Water (10 mL) and EtOAc (5 mL) were added. The

organic layer was separated and the aqueous was re-extracted with EtOAc (5 mL × 3). The

combined organic layers were washed with brine (10 mL), dried over anhydrous Na SO , filtered

2

4

and concentrated in *vacuo*. The residue was purified by reverse column chromatography (25%

ACN in water) to give **int 6** (800 mg, 2.60 mmol, 76% yield) as a yellow oil. MS (ESI) *m/z:*


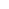


*Supplementary Information*

18

277.0/279.0 (M+H+).

Step

B:

(E)-*tert-*Butyl

(7a-(2,5-difluorophenyl)-3-methyl-4-oxo-6-(1-((2-

(trimethylsilyl)ethoxy)methyl)-1*H*-pyrazol-3-yl)hexahydro-1*H*-pyrrolo[3,4-*d*]pyrimidin-2(3*H*)-

ylidene)carbamate (**int 7**).

To a solution of (E)-*tert*-butyl (7a-(2,5-difluorophenyl)-3-methyl-4-oxohexahydro-1*H*-

pyrrolo[3,4-*d*]pyrimidin-2(3*H*)-ylidene)carbamate **int 1** (70 mg, 0.184 mmol) and **int 6** (51.0

mg, 0.184 mmol) in toluene (2 mL) was added sodium *tert*-butoxide (35.4 mg, 0.368 mmol), 2-

(di-*tert*-butyl phosphino)biphenyl (10.98 mg, 0.037 mmol) and Pd (dba) (16.9 mg, 0.018 mmol)

2

3

under N atmosphere. The mixture was stirred at 70 °C for 2.5 h under N atmosphere. LCMS

2

2

showed desired product mass. The mixture was diluted with water (5 mL) and extracted with

EtOAc (5 mL × 3). The combined organic layers were washed with brine (5 mL), dried over

anhydrous Na SO , filtered, and concentrated in *vacuo* to give the crude product which was

2

4

purified by preparative-TLC (SiO_2_, Pet. ether/EtOAc = 2:1) to afford **int 7** (48 mg, 0.083 mmol,

45.2% yield) as a yellow oil. MS (ESI) *m/z* 577.2 (M+H+).

Step C: (E)-*tert*-Butyl (6-(4-bromo-1-((2-(trimethylsilyl)ethoxy)methyl)-1*H*-pyrazol-3-yl)-7a-

(2,5-difluorophenyl)-3-methyl-4-oxohexahydro-1*H*-pyrrolo[3,4-d]pyrimidin-2(3*H*)-

ylidene)carbamate (**int 8**).

To a stirred solution of **int 7** (50 mg, 0.087 mmol) in DMF (1mL) at 20 °C was added NBS (15.43

mg, 0.087 mmol) under N_2_ atmosphere and the resulting mixture was stirred at 20 °C for 2 h.

LCMS showed major desired product. The mixture was diluted with water (5 mL) and extracted

with EtOAc (5 mL × 3). The combined organic layers were washed with brine (5 mL), dried over

anhydrous Na SO , filtered and concentrated in *vacuo* to give the crude product, which was

2

4

purified by Prep-TLC (SiO_2_, Pet. ether/EtOAc = 1:1) to give **int 8** (26 mg, 0.040 mmol, 45.7%

yield) as a yellow oil. MS (ESI) *m/z* 655.1/657.1 (M+H+).

Step

D:

(E)-*tert*-Butyl

(7a-(2,5-difluorophenyl)-3-methyl-4-oxo-6-(4-phenyl-1-((2-

(trimethylsilyl)ethoxy)methyl)-1*H*-pyrazol-3-yl)hexahydro-1*H*-pyrrolo[3,4-*d*]pyrimidin-2(3*H*)-

ylidene)carbamate (**int 9**).

To a solution of **int 8** (15 mg, 0.023 mmol) in dioxane (1 mL) and water (0.2 mL) was added

K CO (6.32 mg, 0.046 mmol), PdCl (dppf) (1.674 mg, 2.288 µmol) and phenylboronic acid

2

3

2

(3.35 mg, 0.027 mmol), and the mixture was stirred at 75 °C for 2 h under N_2_ atmosphere. LCMS

*Supplementary Information*

19

showed desired product mass. The mixture was diluted with water (5 mL) and extracted with

EtOAc (5 mL × 3). The combined organic layers were washed with brine (5 mL), dried over

Na SO , filtered, and concentrated in vacuo to give the crude product which was purified by Prep-

2

4

TLC (SiO_2_, Pet. ether/EtOAc = 2:1) to give **int 9** (6.0 mg, 9.19 µmol, 40.2% yield) as a yellow

oil. MS (ESI) *m/z* 653.3 (M+H+).

Step E: 7a-(2,5-Difluorophenyl)-2-imino-3-methyl-6-(4-phenyl-1*H*-pyrazol-3-yl)hexahydro-1*H*-

pyrrolo[3,4-*d*]pyrimidin-4(4a*H*)-one (*R/S*-WM447, **28**).

To a stirred solution of **int 9** (6.0 mg, 9.19 µmol) in CH Cl (3 mL) was added TFA (1.0 mL) and

2

2

the resulting mixture was stirred at 20 °C for 3 h. Solvent was removed under reduced pressure.

The mixture was added acetonitrile (2 mL), and NH ·H O (1.5 mL). The mixture was stirred at

3

2

20 °C for 0.5 h. LCMS showed major desired product. Solvent was removed under reduced

pressure. The residue was purified by preparative HPLC (Agela DuraShell C18, 150 × 25 mm, 5

µm; Conditions: water (0.04% NH / H O, 10 mM NH HCO )-ACN; Begin B: 30; End B: 60;

3

2

4

3

Gradient time: 10 min; 100% B Hold time: 2 min; Flow rate: 25 mL/min) to give *R/S*-WM447

(**28**) as a white solid. MS (ESI) *m/z* 423.1 (M+H+). ^1^H NMR (400 MHz, methanol-d_4_) δ 7.57 (s,

1H), 7.42 (br d, *J* = 7.2 Hz, 2H), 7.33 (t, *J* = 7.2 Hz, 2H), 6.98 - 7.24 (m, 4H), 3.85 (d, *J* = 10.4

Hz, 1H), 3.58 - 3.76 (m, 3H), 3.52 (dd, *J* = 2.4, 10.4 Hz, 1H), 3.20 (s, 3H).

**References**

[1]

Panjikar, S., Parthasarathy, V., Lamzin, V.S., Weiss, M.S. and Tucker, P.A. (2005). Auto-

rickshaw: an automated crystal structure determination platform as an efficient tool for

the validation of an X-ray diffraction experiment. Acta Crystallogr. D Biol. Crystallogr.

61, 449-457.

[2]

[3]

[4]

Emsley, P. and Cowtan, K. (2004). Coot: model-building tools for molecular graphics.

Acta Cryst. D. Biol. Crystallogr. 60, 2126-2132.

Adams, P.D. et al. (2010). PHENIX: a comprehensive Python-based system for

macromolecular structure solution. Acta Cryst. D. Biol. Crystallogr. 66, 213-221.

Hodder, A.N. et al. (2022). Basis for drug selectivity of plasmepsin IX and X inhibition

in *Plasmodium falciparum* and *vivax*. Structure 30, 947-961 e6.

Referee: 1

Comments for Transmission to Authors

The paper by Hodder et al. presents an SAR study on a series of molecules

targeting plasmodium proteases called plasmepsins (PMX). There are 10

plasmepsins, of which PMX V and X appear to be the most important ones for

microbial viability. Therefore the ultimate goal is to generate dual PMV/PMX

inhibitors.

The authors have identified a core pharmacophore called the IPF scaffold, of which

two (WM-36 and WM-48 were the most potent in vitro) and embarked on an SAR

study examining in vitro and in vivo activity. The most promising compounds were

subjected to structural analyses.

The paper is very well written. Methods are described in much detail.

Major points

Neither WM-36 and WM-48 have in vivo activity, despite WM-48 inhibiting PMXV, IX,

and X with nanomolar activity. In that regard, the statement on Page 16, line 34 “it is

assumed that the … PMX X activity of WM-48 was just beyond the cusp of activity” is

hand-waving. An alternative interpretation would be that potency and selectivity are

insufficient predictors of in vivo activity, but this possibility has not been addressed.

Supporting this alternative interpretation is the fact that their best analog WM-396

and compounds 24 and 26 in Table 2 have antiparasitic activity, despite having

similar in vitro potencies than WM-36 and WM-48.

Authors response: WM-396 has potent inhibition of PMX whereas WM36 and WM48

do not. We have altered the text as below to clarify this point, that the biochemical

PM and parasite correlation suggest that the antiparasitic activity observed is derived

from inhibition of PMX.

‘The PMX inhibitory activity of S-WM48 (14) was not sufficiently potent to register

antiparasitic activity at concentrations below 10 µM, whereas compounds 24 - 26

that exhibit potent inhibition of PMX (IC50 <0.10 µM) registered anti-parasitic activity

(Table 2). Moreover, the level of PMV inhibition by S-WM48 was not adequate to

register antiparasitic activity. In comparison, WEHI-842 (1) and WEHI-601 (2) that

exhibit potent inhibition of PMV (IC50 <0.02 µM) exhibit antiparasitic activity (Fig. 1).

Collectively, these data suggest that the antiparasitic activity observed with analogs

24 – 26 and 28 is derived from the inhibition of PMX.’

A series of substitutions on the biaryl side chain of the core structure has revealed

information about the S1 and S2 pockets on the roof of the catalytic cavity.

Comparative binding studies of the hit compound(s) WM36 and WM48 to the most

potent PMX inhibitors WM396 and WM447 further suggested additional areas of

PMX and PMV that could be further exploited by targeted modification of the

molecule. How exactly this would be achieved has, however, only vaguely

addressed.

Authors response: This is discussed in the discussion and is graphically represented

in the Supplementary figures S2-S5.

A few minor points.

The graphical representations of structures in Figures are hard to read and dissect.

The dark grey and blue ribbons are quite prominent and seem to obscure binding

modes. Modeled compounds are quite small and colors chosen make it difficult to

discern differences and similarities (for example, Fig 6 shows one compound in red

and the other in orange). Can figures be made larger? and clearer by more

prominently displaying important points of interactions as discussed in the text?

Authors response: The structure figures have been redone for Fig. 3, 4, 5, 6, 7 and 8

to make the colours and the shading easier to see and interpret. Figures have been

enlarged where possible.

The sentences on page 17 lines 19-23 and 30-33 , and page 24, lines 50-55 seem

odd. Please check grammar.

Authors response: Page 24, lines 50-55 could not be identified as there are not that

many lines in the page. This page and the full document has been checked and

changed as needed. However, the document has been extensively edited to ensure

all sentences make sense. The grammar has also been corrected where identified.

Page 16, line 34. There are some compound identifiers that have not been defined.

Authors response: This has been corrected as below.

‘The PMX inhibitory activity of S-WM48 (14) was not sufficiently potent to register

antiparasitic activity at concentrations below 10 µM, whereas compounds 24 - 26

that exhibit potent inhibition of PMX (IC50 <0.10 µM) registered anti-parasitic activity

(Table 2). Moreover, the level of PMV inhibition by S-WM48 was not adequate to

register antiparasitic activity. In comparison, WEHI-842 (1) and WEHI-601 (2) that

exhibit potent inhibition of PMV (IC50 <0.02 µM) exhibit antiparasitic activity (Fig. 1).

Collectively, these data suggest that the antiparasitic activity observed with analogs

24 – 26 and 28 is derived from the inhibition of PMX.’

Page 18, line 58. What is a “statine”?

Authors response: Statine is a modified amino acid that is a well-described aspartyl

protease inhibitor that was first described as a component of the natural product

pepstatin. Statine has been incorporated in the peptidomimetic PMV inhibitors by

Sleebs et al. cited within. An explanation has been added to the text as below.

‘Although the IP head group of WM48 and the hydroxy moiety of the statine (a

modified amino acid that is an aspartyl protease inhibitor {Sleebs, 2014 #13350}) in

WEHI-842 both interact with the active site aspartyl residues (D80 and D313) of

PvPMV, their overall interactive surfaces are different.’

Referee: 2

Comments for Transmission to Authors

The authors generated different substituents of the fused pyrrolidine imino

pyrimidinones (IPF) and their potential inhibition properties against Plasmepsin V, IX

and X, estimated their antimalarial potential, and subsequently analyzed their

structure-activity relationship through crystallography to explore binding modes and

selectivity, aiming to develop novel antimalarial therapeutics. Although the research

was thoroughly conducted, there are significant concerns for rational ground,

presentation, and writing.

1. In the antimalarial drug discovery, any compound inhibiting host enzymes is not

preferred; therefore, screening a library of 1,298 compounds that were known to

inhibit human aspartyl proteases is not rational. A justification needs to be included

in the manuscript.

Authors response: Repurposing compounds that have been discovered in drug

discovery programs for new treatments against human disease to treat neglected

disease is a common approach in drug discovery. Indeed, both WM382 and

UCB7362 which are potent plasmepsin inhibitors were discovered using this

approach. WM382 is a lead compound that was developed from the initial 32 hit

compounds identified by screening the library of 1,298 compounds as we have

described previously (Favuzza et al., Cell Host and Microbe 2020). MK7602, an

optimised derivative of WM382 is now in human clinical trials for treatment of malaria

demonstrating this approach is successful. We have expanded the description of

WM382 and UCB7362 in the introduction and results to better explain this approach

as below.

‘WEHI-842 and WEHI-601 have modest activity against the asexual parasite despite

their potent biochemical inhibition of PMV (Fig. 1) which was thought to be attributed

to their low membrane permeability [33,34]. However, recent evidence suggests that

PMV is highly expressed and therefore concentrations of an inhibitor more than its

enzymatic IC50 value may be required to enable efficient killing of the malaria

parasite [35]. A further limitation of using peptidomimetics to target PMV is their low

metabolic stability which hinders their development as antimalarial therapeutics. To

uncover drug-like starting points of plasmepsins, independent screens of human

aspartyl protease inhibitor libraries against the P. falciparum were undertaken and

uncovered starting points which were then developed into WM382 which potently

targets both PMIX and PMX [18,20] and UCB7362 more specifically targets PMX

[21] (Figure 1). To date no drug-like compounds have been discovered that potently

target PMV.’

‘To uncover non-peptidomimetic drug-like inhibitors of the aspartic protease PvPMV,

we screened a library of 1,298 compounds that were known to inhibit human aspartyl

proteases [36-39]. These compounds were selected by chemoinformatic structural

diversity from a larger library of compounds that targeted human aspartyl proteases

and has been used previously to identify hit compounds that inhibited P. falciparum

growth [18]. These hit compound belonged to a single chemical class and were used

to develop a lead compound WM382 that is a potent dual selective inhibitor of PMIX

and PMX from P. falciparum and P. vivax [18,49]. The screen utilized a FRET-based

assay with PvPMV and a fluorogenic EDANS-DACBYL labeled peptide with the P.

falciparum knob associated histidine-rich protein (KAHRP) PEXEL motif amino acid

sequence. This screen identified the hit compound WM36 (8) and a dose-response

assay against PMV confirmed the inhibitory activity with an IC50 of 0.807 µM (Table

1).’

2. The rationale behind selecting different groups for substitution reactions needs to

be given.

Authors response: We have given more detailed explanation for each of the

transnational changes made to the scaffold in Tables 1, 2 and 3 and Figure 2 in each

of the paragraphs in the structure activity relationship section.

‘Examination of the SAR began with point modifications with small functional groups

on the terminal aryl ring of the biphenyl motif to determine whether these changes

are favourable or deleterious for the inhibition of PMV, PMIX or PMX.’

‘The inclusion of a second substituent on the terminal aryl ring may give an

additional interaction with amino acids in the S1 pocket leading to enhanced

inhibition of PMV, PMIX or PMX. This first combination of analogs had a 4-chloro

while altering the 2-position with a small functional group.’

‘The introduction of a thiophene ring would slightly alter the substituent vector bond

angle compared to substitution on the aryl ring which could be favorable for the

inhibition of PMV, PMIX or PMX or selectivity between these proteases.‘

‘The fused pyrrolidone group for each isomer would be orientated in a different

direction and therefore one isomer should be preferred for binding to the S1’ and S1

pocket of the plasmepsins.’

3. S-WM 48 could inhibit PMV IX and X but does not have antimalarial properties.

The observation justification is confusing and needs to be revised.

Authors response: We have revised the text describing the relationship between the

plasmepsins and antiparasitic activity in the results as below to make it less

confusing.

‘It was observed that the S-WM48 (14) exhibited potent inhibition of PMV, PMIX, and

PMX (IC50s 0.096, 0.390 and 0.133 µM), while R-WM48 (31) had significantly

reduced activity against PMV, PMIX, and PMX (IC50s >10, >10, and 1.0 µM) (Fig.

2). S-WM48 (14) did not exhibit antimalarial activity at the concentration tested

(EC50 >10 µM). The PMX inhibitory activity of S-WM48 (14) was not sufficiently

potent to register antiparasitic activity at concentrations below 10 µM, whereas

compounds 24 - 26 that exhibit potent inhibition of PMX (IC50 <0.10 µM) registered

anti-parasitic activity (Table 2). Moreover, the level of PMV inhibition by S-WM48

was not adequate to register antiparasitic activity. In comparison, WEHI-842 (1) and

WEHI-601 (2) that exhibit potent inhibition of PMV (IC50 <0.02 µM) exhibit

antiparasitic activity (Figure 1). Collectively, these data suggest that the antiparasitic

activity observed with analogs 24 – 26 and 28 is derived from the inhibition of PMX.’

4. The manuscript does not mention experimental replicates, controls, or methods for

managing data variability. Clearly state the use of positive and negative controls, the

number of replicates, and the statistical methods employed.

Authors response: We have included these details into the experimental section as

well as figure legends where needed. The replicate details are also mentioned in the

footnote of each table.

‘Plasmepsin V, IX, and X fluorogenic assays

These fluorogenic assays follow protocols previously described [18,40,41].

Compound potency was assessed with 10 point, 3-fold dilution series starting at

100µM in duplicate per experiment and repeated in three independent experiments.

Data were normalized to percent inhibition relative to 1% DMSO (high control) and

either 1µM WEHI-600 for PMV and 1µM WM382 for PMIX/PMX (low controls). IC50

values (relative inflection of dose response curve) were calculated using a nonlinear

regression four-parameter fit analysis in Dotmatics 5.3 and Spotfire 7.11.1 software.

The equation used was sigmoidal dose response (variable slope), Y = bottom + (top

− bottom)/(1+10 ((logIC50 − X) × Hill Slope)). Reported IC50 values were calculated

based on the averages of three independent dataset. Assay robustness and data

quality was assessed by Z’ >0.5 (Z’= 1-(3*(SD high + SD low)/(mean high – mean

low))) and activity reproducibility of reference compound. WEHI-600 (PMV) or

WM382 (PMIX/PMX) were included as reference compounds in every experiment to

monitor the assay performance. Any drift in activity >3 fold from the average

acceptable range and the experiment was rejected and repeated.

P. falciparum growth inhibition assays

The ability of compounds to block growth of blood stage P. falciparum parasites was

determined using a growth inhibition assay (GIA) as described [18]. Compound

potency was assessed with 10 point, 3-fold dilution series starting at 10µM in

duplicate per experiment and repeated in three independent experiments. Data were

normalized to percent viability relative to 0.1% DMSO (high growth control) and 2.5

µM chloroquine (low growth control). EC50 values (relative inflection of dose

response curve) were calculated using a nonlinear regression four-parameter fit

analysis in Dotmatics 5.3 and Spotfire 7.11.1 software. The equation used was

sigmoidal dose response (variable slope), Y = bottom + (top − bottom)/(1+10

((logEC50 − X) × Hill Slope)). Reported EC50 values were calculated on the average

of three independent dataset. Assay robustness and data quality was assessed by Z’

>0.5 (Z’= 1-(3*(SD high + SD low)/(mean high – mean low))) and activity

reproducibility of reference compound WM382 which was included in every

experiment. Any drift in WM382 activity >3 fold from the average acceptable range

and the experiment was rejected and repeated.’

‘Fig. 2. Plasmepsin biochemical and P. falciparum asexual IC50 and EC50 values of

the racemic mixture (R/S), and S and R-stereoisomers of WM48. Data are averages

(SDs) of n=3 experiments.’

5. Tables, particularly Table 3, need formatting to meet publication quality standards.

Authors response: The Table formatting has been checked and altered to ensure it

adheres to journal guidelines.

6. Some figure legends, such as for Fig. 1 and 2, are unclear. Improve the legends to

convey the correlation between structural modifications and biological activities.

Authors response: The figure legends have been updated as below.

‘Fig. 1. Structures and biological activities of plasmepsin V, IX, and X inhibitors from

literature. Shown are the Plasmepsin aspartic protease activity (IC50) for PMV, PMIX

and PMX. Also shown is the blood stage inhibitory growth activity (EC50) of each

compound against the 3D7 strain of P. falciparum 3D7 obtained from the literature

[17, 18, 21, 33, 34].’

‘Fig. 2. Inhibition of the proteases PMV, PMIX and PMX (IC50) and P. falciparum

blood stage growth (EC50) by the racemic mixture (R/S), and S and R-stereoisomers

of WM48. Data are averages (SDs) of n=3 experiments.’

‘Fig. 4. Graphical representation and comparison of the structures for PvPMV-WM36

and PvPMV-WM48. (A) Front view showing a cartoon of the entire PvPMV-WM36

complex. Box shows magnification area for (B) and (C). (B) Magnified front view with

WM36 shown in green, active site aspartic acid residues (D80 and D313) highlighted

in magenta, and PvPMV in grey. (C) Side view (90˚ anti clockwise rotation about the

vertical axis of (B)) with the position of 2-chlorine atom of the chlorobenzene moiety

(Wheat) indicated by the arrow. Other structural features for orientation include the

active site D80 and D313 (magenta) and the S2 Flap which covers the front of the

catalytic cleft. (D) Front view showing a cartoon of the entire PvPMV-WM48

complex. Box shows magnification area for (E) and (F). (E) Magnified front view with

WM48 shown in cyan, active site aspartic acid residues (D80 and D313) highlighted

in magenta, and PvPMV in grey. (F) Side view (90˚ anti clockwise rotation about the

vertical axis of (E)) with the position of para chlorine atom of the chlorobenzene

moiety (Wheat) indicated by the arrow. Other structural features for orientation

include the active site D80 and D313 (magenta) and the S2 flap.’

7. The manuscript mentions other inhibitors like WM382 but does not adequately

compare the efficacy and selectivity of the IPF analogues against these existing

inhibitors. A direct comparison of efficacy, stability, and resistance profiles is

necessary.

Authors response: The IPF analogues are in the early stage of exploration and their

antimalarial potencies do not warrant metabolic stability, mouse efficacy and

resistance profiling. This will be performed for IPF compounds as they are developed

further.

8. On page 11, the table "LCMS and 1H-NMR for Representative Final Compounds"

should be appropriately labelled, numbered, and placed in the designated table

section.

Authors response: The chemistry experimental has been relocated into the

Supplementary Section. Figures outlining the synthesis have been added to detail

the compound numbering more clearly.

9. Different moieties of the primary compounds, such as IPF, undergo substitution

and need to be presented in different colours for better understanding.

Authors response: The R-group on the compound structure associated with each

Table distinguishes where changes have been made. The stereochemistry changes

in Figures indicates the differences between compounds. We would prefer not to

incorporate colour.

Colors have been used in the appropriate figures to highlight the moieties involved in

interactions with the PMV and PMX surfaces.

10. The discussion section is overly detailed and has unnecessary information;

hence, it should be condensed to no more than one page, reducing the current

length (pages 21-26).

Authors response: The discussion has been shortened considerably from the original

submission. Whilst we have not reduced it to one page it is at least half what it was

and would prefer to not reduce it further.

11. The discussion would be stronger if findings were integrated with the broader

context of plasmepsin inhibitors. Relating these findings to existing knowledge about

plasmepsin inhibition and malaria drug development could provide a more

comprehensive understanding of their significance. Further, it should highlight the

significance of WM36 and its analogues in inhibiting PMV, PMIX, and PMX,

emphasizing their unique binding interactions compared to WM4 and the need for

further refinement to enhance their dual inhibition potential. It should analyze the

impact of structural modifications, such as the biphenyl moiety and its 4-chloro

substitution in WM48, stereochemistry favouring S-isomers, and the influence of

thiophene and other substituents on enzyme inhibition. Structural insights gained

from crystal structures should be discussed, especially how minor substituent

changes affect binding affinity and interactions in the PMV and PMX binding pockets.

The gap between enzyme inhibition and antiparasitic activity should be addressed,

stressing the need for additional structural and biological evaluations to improve

efficacy against the parasite. The discussion should suggest exploring modifications

to hydrophilic clusters in the S2' pocket of PMX and PMV to enhance inhibitor

potency and selectivity, guiding the refinement of these analogs for optimized

therapeutic outcomes.

Authors: Each point in 11 has been broken up into a), b), c), d) and e) and

addressed individually below.

a). Relating these findings to existing knowledge about plasmepsin inhibition and

malaria drug development could provide a more comprehensive understanding of

their significance

Authors response: The introduction and discussion have been modified.

b). Further, it should highlight the significance of WM36 and its analogues in

inhibiting PMV, PMIX, and PMX, emphasizing their unique binding interactions

compared to WM4 and the need for further refinement to enhance their dual

inhibition potential.

Authors response: Figure 7 shows the comparison of the predicted interactive areas

of WM48with pmx which has been compared to the interactive sites of WM4 and

WM842 in pmx. Figure 6(E) shows the different effects on protein conformation

generated by WM48 in comparison with one of the most potent PMV inhibitors,

WEHI842. A new figure 6(E) discusses the relative change in residue positions in

these different structures that result in the opening and closing of cavity induced by

positioning of the inhibitor.

c). It should analyze the impact of structural modifications, such as the biphenyl

moiety and its 4-chloro substitution in WM48, stereochemistry favouring S-isomers,

and the influence of thiophene and other substituents on enzyme inhibition.

Authors response: The influence of the substitutions for WM48, WM396 (thiophene)

and WM447 (pyrazole) are discussed structurally in terms of important binding

interactions and in relation to the local cavity surface. See modified Supplementary

figs2-5. A comment on the occurrence of the of S-isomer structure for high affinity

binding relative to the R-isomer in the results.

d). Structural insights gained from crystal structures should be discussed, especially

how minor substituent changes affect binding affinity and interactions in the PMV

and PMX binding pockets

Authors response: This is the basis of the discussion around the structures for the

PvPMV-WM36 and WM48 structural comparisons. The S2-5 figures compare

predicted differences for inhibitor binding in pmv and pmx.

e). The discussion should suggest exploring modifications to hydrophilic clusters in

the S2' pocket of PMX and PMV to enhance inhibitor potency and selectivity, guiding

the refinement of these analogs for optimized therapeutic outcomes.

Authors response: This is now in the discussion with reference to Fig S6

12. A graphical abstract is required to comprehend the study.

Authors response: We have now provided a graphical abstract.
